# Social calls influence the foraging behavior in wild big-footed myotis

**DOI:** 10.1186/s12983-020-00384-8

**Published:** 2021-01-07

**Authors:** Dongge Guo, Jianan Ding, Heng Liu, Lin Zhou, Jiang Feng, Bo Luo, Ying Liu

**Affiliations:** 1 Jilin Provincial Key Laboratory of Animal Resource Conservation and Utilization, Northeast Normal University, 2555 Jingyue Street, Changchun, 130117 China; 2 College of Life Science, Jilin Agricultural University, 2888 Xincheng Street, Changchun, 130118 China; 3 Key Laboratory of Southwest China Wildlife Resources Conservation of Ministry of Education, China West Normal University, Nanchong, 637002 China

**Keywords:** Bats, Social calls, Foraging, *Myotis macrodactylus*, Dietary analysis, Food competition

## Abstract

**Background:**

Why a variety of social animals emit foraging-associated calls during group foraging remains an open question. These vocalizations may be used to recruit conspecifics to food patches (i.e. food advertisement hypothesis) or defend food resources against competitors (food defence hypothesis), presumably depending on food availability. Insectivorous bats rely heavily on vocalizations for navigation, foraging, and social interactions. In this study, we used free-ranging big-footed myotis (*Myotis macrodactylus* Temminck, 1840) to test whether social calls produced in a foraging context serve to advertise food patches or to ward off food competitors. Using a combination of acoustic recordings, playback experiments with adult females and dietary monitoring (light trapping and DNA metabarcoding techniques), we investigated the relationship between insect availability and social vocalizations in foraging bats.

**Results:**

The big-footed myotis uttered low-frequency social calls composed of 7 syllable types during foraging interactions. Although the dietary composition of bats varied across different sampling periods, Diptera, Lepidoptera, and Trichoptera were the most common prey consumed. The number of social vocalizations was primarily predicted by insect abundance, insect species composition, and echolocation vocalizations from conspecifics. The number of conspecific echolocation pulses tended to decrease following the emission of most social calls. Feeding bats consistently decreased foraging attempts and food consumption during playbacks of social calls with distinctive structures compared to control trials. The duration of flight decreased 1.29–1.96 fold in the presence of social calls versus controls.

**Conclusions:**

These results support the food defence hypothesis, suggesting that foraging bats employ social calls to engage in intraspecific food competition. This study provides correlative evidence for the role of insect abundance and diversity in influencing the emission of social calls in insectivorous bats. Our findings add to the current knowledge of the function of social calls in echolocating bats.

**Supplementary Information:**

The online version contains supplementary material available at 10.1186/s12983-020-00384-8.

## Background

Numerous animals live in social groups, ranging from insects and fishes to birds and mammals [1–3]. Group living provides multiple benefits such as social information transfer [4, 5], group defence against predators [6], and efficient thermoregulation [7]. However, living in large groups also incurs some costs, including intense competition for limited food resources, increased predation risk due to conspicuousness, and high probability of disease transmission [8, 9]. The social foraging theory emphasizes that animals’ foraging behavior can be largely influenced by the social environment, e.g., individual energetic gains are determined by the presence of foraging conspecifics [10, 11]. In some cases, joining a foraging group largely improves the efficiency of detecting and capturing the prey [12]. When the group becomes too dense, increased competition for food and conspecific interference will elicit intraspecific agonistic interactions, which in turn reduce foraging efficiency of group members [13–15].

Food-associated calls are social vocalizations produced by gregarious animals during foraging [16]. These vocalizations are usually loud, low frequency bouts facilitating long-range transmission of information [17]. The food advertisement hypothesis proposes that the sender emits calls to inform conspecifics about food availability, eliciting conspecific aggregation and cooperative foraging [18–21]. Information transfer concerning food sources reduces the time spent searching for food and therefore enhances foraging efficiency of relatives and non-relatives. For instance, naked mole-rats (*Heterocephalus glaber*) utter conspicuous vocalizations to recruit their mates when detecting new food patches [18]. In rhesus macaques (*Macaca mulatta*), food-associated calls encode information on the types of food, and group members rapidly approach the sound source upon hearing the functionally referential signals [19, 20]. Similarly, the squeak calls from colonially nesting cliff swallows (*Hirundo pyrrhonota*) attract conspecifics to feed on ephemeral insect swarms [21]. In these circumstances, the number of food-associated calls is predicted to be positively related to food availability and conspecifics’ foraging activities. In contrast, the food defence hypothesis underscores that the sender utter calls to warn off potential competitors from feeding areas, resulting in a decreased likelihood of agonistic encounters and physical injury [22–25]. The food defence hypothesis predicts that the number of food-associated calls is negatively associated with food availability and foraging activities of group members [25]. Support for the food defence hypothesis has been found in some birds and primates, especially for those foraging in large groups. For instance, the group-living green woodhoopoes (*Phoeniculus purpureus*) employ kek calls to monopolize the richest food patches [22]. The chuck calls given by pied babblers (*Turdoides bicolor*) serve to regulate the spacing between foragers, and playback of chuck calls causes the competitors to move away from the speaker [23]. In white-faced capuchins (*Cebus capucinus*) and red-bellied tamarins (*Saguinus labiatus*), individuals who called when they discovered food were less likely to be approached by other competitors [24, 25].

Insectivorous bats are an interesting group to study social function of food-associated calls. Most insectivorous bats dwell gregariously in natural or man-made shelters and initiate foraging activities after sunset [26, 27]. They not only emit echolocation pulses to navigate and search for insects but also use social calls to cope with intraspecific interactions [28–30]. Previous studies have shown that some bats search for food by eavesdropping on echolocation vocalizations from group members [31–34]. However, the role of bat echolocation signals in long-range information transfer appears to be limited, given that high-frequency sounds are subject to strong atmospheric attenuation with distance [35]. Indeed, greater spear-nosed bats (*Phyllostomus hastatus*) produce low-frequency screech calls at a higher rate when feeding on concentrated food resources, thereby coordinating foraging activities of conspecifics [36]. Some bat foragers, however, emit loud and repetitive social calls to warn potential competitors away from food patches under limited food conditions. Examples include the common pipistrelles *Pipistrellus pipistrellus* [37], big brown bats *Eptesicus fuscus* [38], and Asian particoloured bats *Vespertilio sinensis* [30]. In Mexican free-tailed bats (*Tararida brasiliensis*), senders even deliberately emit sinusoidal frequency-modulated (SFM) calls to compete for food by jamming the echolocation of conspecifics [39]. These findings indicate that the function of social vocalizations in foraging bats may differ by species. Nonetheless, previous studies are subject to two limitations: (1) the dietary composition of bats studied has not been considered, yielding potential bias in quantifying the relationship between bat social calls and insect abundance; and (2) since food competition also depends on the relative abundance of preferred prey [37], social calling behavior in foraging bats may be affected by the diversity of insects (e.g., insect evenness), albeit empirical test of this idea is lacking.

The aim of this study was to investigate the function of social calls in the context of feeding in the wild big-footed myotis (*Myotis macrodactylus* Temminck, 1840). In particular, we tested whether social calls emitted by foraging big-footed myotis function to recruit conspecifics to food patches or to defend food resources. *Myotis macrodactylus* is a trawling forager that captures insects from or above the water surface [40, 41]. These bats are distributed in the northeast of China, Korea, Japan and Russia [42]. Adult females form maternal colonies composed of thousands of members and emerge to foraging soon after local sunset [40]. These bats forage in groups and emit diverse social vocalizations [43]. To achieve our goal, we applied next generation sequencing of fecal DNA to identify the dietary composition of *M. macrodactylus*, and monitored bat social vocalizations as a function of insect prey abundance and evenness in the foraging area. We recorded bat echolocation vocalizations before and after the emission of social calls to determine whether social calls can attract or repel conspecifics [27]. We also conducted playback experiments to determine whether adult females initiated different foraging activities when exposed to experimental stimuli, echolocation pulses, and silence. If the food advertisement hypothesis accounts for the function of social calls in *M. macrodactylus*, we predict that the number of bat social vocalizations would be positively associated with insect abundance and evenness. The number of bat echolocation pulses would increase after the emission of social calls. The foraging activity (i.e. food consumption and flight duration) of adult females would be enhanced during playback of social vocalizations versus playback of controls (i.e., silence and echolocation calls). If the food defence hypothesis holds for this bat species, we expect that individuals would increase the emission of social vocalizations when insect abundance and evenness are comparatively low. The number of bat echolocation vocalizations would decrease after the output of social calls. The feeding bats would reduce foraging attempts and food consumption in the presence of experimental stimuli.

## Results

### Characteristics of social vocalizations

**Fig. 1:**
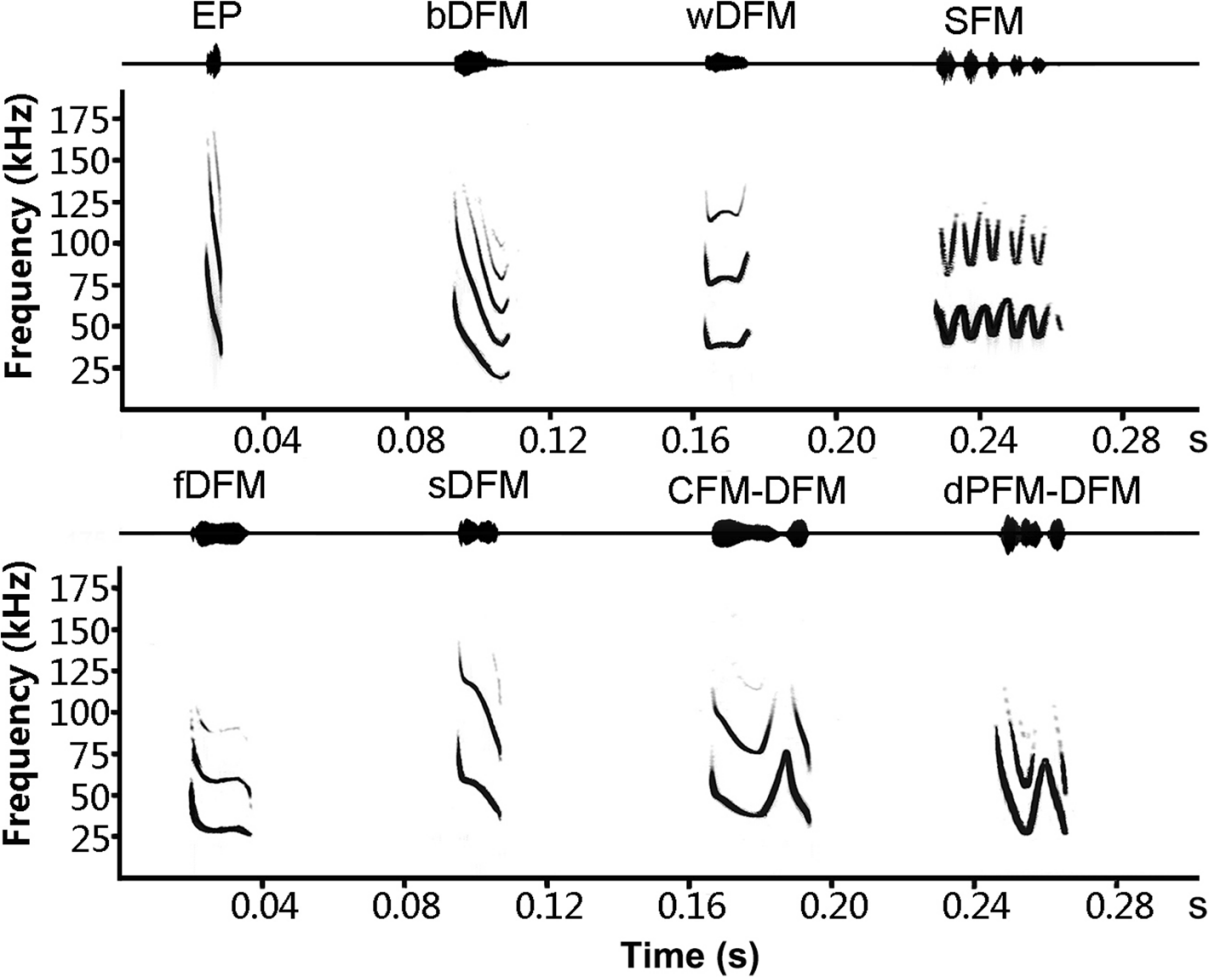
Waveforms (upper trace) and spectrograms (lower trace) of echolocation and social vocalizations in foraging big-footed myotis. EP: search-phase echolocation pulse. bDFM: bent downward frequency modulation. wDFM: wrinkled downward frequency modulation. SFM: sinusoidal frequency modulation. sDFM: steep downward frequency modulation. fDFM: flattened downward frequency modulation. CFM-DFM: chevron frequency modulation-downward frequency modulation. dPFM-DFM: downward paraboloid frequency modulation-downward frequency modulation

A total of 3209 syllables belonging to 7 types were obtained in the focal foraging site of *M. macrodactylus*, namely bent downward frequency modulation (bDFM), wrinkled downward frequency modulation (wDFM), sinusoidal frequency modulation (SFM), flattened downward frequency modulation (fDFM), steep downward frequency modulation (sDFM), chevron frequency modulation-downward frequency modulation (CFM-DFM), and downward paraboloid frequency modulation-downward frequency modulation (dPFM-DFM) (Fig. 1). All syllables contained the highest energy in the first harmonic. The duration of syllables ranged from 9.79 to 54.18 ms, and peak frequency ranged from 25.13 to 50.50 kHz. bDFM was the most commonly observed syllable, followed by wDFM and sDFM. bDFM had a relatively long duration (17.14 ± 2.74 ms), low frequency (37.25 ± 3.51 kHz) and large bandwidth (35.19 ± 7.34 kHz). bDFM was emitted in sequence at a high repetition rate. wDFM was an irregular wrinkling frequency modulated syllable. The duration and peak frequency of wDFM were 18.54 ± 2.74 ms and 39.14 ± 5.69 kHz, respectively. SFM was composed of upward and downward frequency–modulated segments, with a long duration (38.18 ± 6.86 ms) and high frequency (48.34 ± 7.93 kHz). SFM was always emitted along with echolocation pulses in both search and terminal phases.

The paired samples *t*-test indicated that emission of bDFM and fDFM calls caused a significant decrease in the number of echolocation pulses among conspecifics (bDFM: number of echolocation pulses before calls = 5.53 ± 0.56, number of echolocation pulses after calls = 4.94 ± 0.56, mean differences = 0.59, *t* = 2.12, *df* = 215, *P* = 0.036; fDFM: number of echolocation pulses before calls = 8.25 ± 1.94, number of echolocation pulses after calls = 6.22 ± 1.46, mean differences = 2.03, *t* = 2.12, *df* = 31, *P* = 0.039). Similarly, the presence of wDFM, SFM, and CFM-DFM calls also induced a decrease in the number of conspecific echolocation vocalizations, albeit such difference was not statistically significant (all *P* > 0.05; Table 1). By contrast, the presence of sDFM calls induced a marked increase in the number of echolocation vocalizations (Number of echolocation pulses before calls = 2.84 ± 0.48, number of echolocation pulses after calls = 3.52 ± 0.52; mean differences = − 0.68, *t* = − 2.31, *df* = 124, *P* = 0.023; Table 1).

**Table 1:** The number of conspecific echolocation pulses before and after the presence of social calls

Types of signals	N_before_	N_after_	Mean differences	*t*	*df*	*P*
bDFM	5.53 ± 0.56	4.94 ± 0.41	0.59	2.12	215	0.036 *
wDFM	2.93 ± 0.70	2.39 ± 0.53	0.54	1.64	120	0.104
SFM	2.17 ± 0.66	2.10 ± 0.68	0.07	0.09	29	0.927
fDFM	8.25 ± 1.94	6.22 ± 1.46	2.03	2.12	31	0.039 *
sDFM	2.84 ± 0.48	3.52 ± 0.52	−0.68	−2.31	124	0.023 *
CFM-DFM	4.32 ± 0.85	4.66 ± 1.02	−0.34	−0.42	52	0.677
dPFM-DFM	8.49 ± 1.64	7.32 ± 1.25	1.17	1.29	40	0.204

*N*_*before*_ the number of echolocation pulses before the presence of social calls, *N*_*after*_ the number of echolocation pulses after the presence of social calls. N_before_ and N_after_ are given as mean ± SE. Mean differences = N_before_ − N_after_. *bDFM* bent downward frequency modulation, *sDFM* steep downward frequency modulation, *fDFM* flattened downward frequency modulation, *wDFM* wrinkled downward frequency modulation, *SFM* sinusoidal frequency modulation, *CFM-DFM* chevron frequency modulation-downward frequency modulation, *dPFM-DFM* downward paraboloid frequency modulation-downward frequency modulation. Statistical significance is based on the paired-samples *t* test

### Temporal variation in diet

**Fig. 2:**
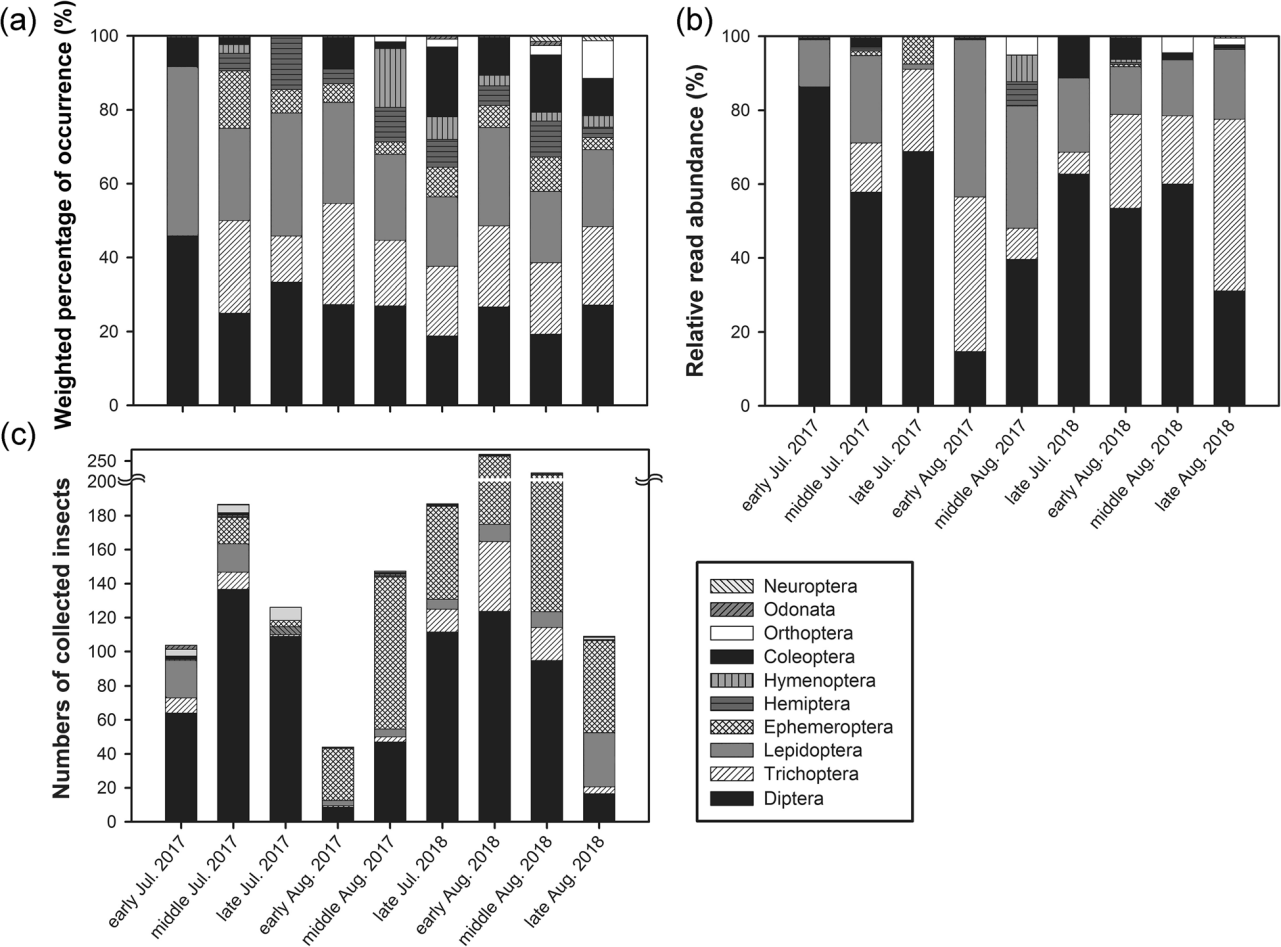
Temporal variation in the diets of *M. macrodactylus* and insect diversity in the focal transect. **a** Weighted percentage of occurrence (wPOO). **b** Relative read abundance (RRA). **c** Insect abundance in the focal transect

We obtained 3.26 million quality-filtered DNA sequences of insect prey from faecal samples, with an average of 45,304 sequences per sample. We identified 444 molecular operational taxonomic units (MOTUs) of prey items in the diet of the big-footed myotis, containing 59 insect families belonging to 10 orders. These insects belonged to the orders Diptera, Trichoptera, Lepidoptera, Ephemeroptera, Hemiptera, Hymenoptera, Coleoptera, Orthoptera, Odonata, and Neuroptera (Table S1, Additional file 1). Diptera were found in the diet of all captured bats, while Lepidoptera and Trichoptera occurred in 95.8 and 84.7%, respectively. The dietary composition of *M. macrodactylus* showed pronounced variation across different sampling periods (weighted percentage of occurrence data, wPOO: *df* = 72, χ^2^ = 275.99, *P* < 0.001; relative read abundance data, RRA: *df* = 72, χ^2^ = 423.03, *P* < 0.001; Fig. 2a, b). There was no significant difference in the diets between males and females (wPOO: *df* = 9, χ^2^ = 9.53, *P* = 0.39; RRA: *df* = 9, χ^2^ = 14.41, *P* = 0.11).

A total of 4788 insect samples from 61 families in 10 orders were collected by light trapping. Each order was found in *M. macrodactylus*’ dietary composition except for Homoptera. Diptera was the most common order (51.32%), followed by Ephemeroptera (31.22%), Trichoptera (7.87%), and Lepidoptera (6.68%) (Fig. 2c; Table S2, Additional file 2). At the order level, the composition of insects from the focal transect was positively related to wPOO (OLS: Estimate = 0.71 ± 0.27, *t =* 2.64, *P* = 0.034, *R*^*2*^ = 0.498) and RRA (OLS: Estimate = 0.74 ± 0.26, *t =* 2.89, *P* = 0.023, *R*^*2*^ = 0.544) of prey in faecal pellets.

### Relationship between food availability and social vocalizations

**Fig. 3:**
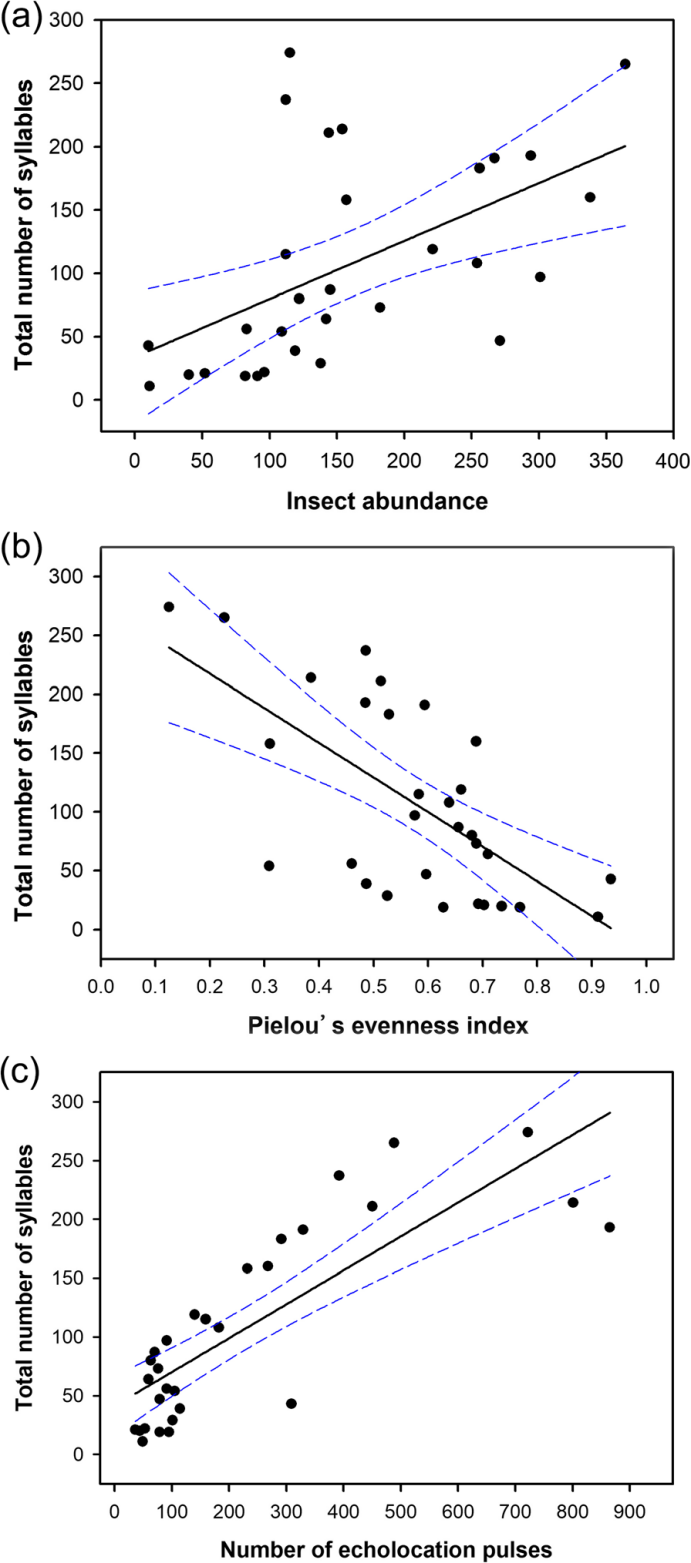
Relationships among bat vocalizations, insect abundance, and insect diversity (*N* = 30). **a** Total number of syllables and insect abundance. **b** Total number of syllables and insect diversity. **c** Total number of syllables and number of echolocation pulses. The solid lines in the middle of the frames represent the regression models, and the dashed lines represent 95% confidence intervals

The insect abundance, evenness index of insects, and number of echolocation pulses influenced the number of social vocalizations in foraging bats (Table S3, Additional file 3). The emission of social calls correlated positively with insect abundance (GLMM: Estimate = 3.89E− 3 ± 1.04E− 3, *t =* 3.76, *P* < 0.001; Fig. 3a; Table 2), but negatively with the evenness index of insects (GLMM: Estimate = − 1.22 ± 0.61, *t =* − 2.01, *P* = 0.048; Fig. 3b; Table 2). The emission of social calls was also correlated positively with the abundance of Diptera (GLMM: Estimate = 9.06E− 3 ± 2.34E− 3, *t* = 3.88, *P* < 0.01), and Trichoptera (GLMM: Estimate = 1.84E− 2 ± 9.48E− 3, *t* = 1.94, *P* = 0.052). There was a negative correlation between the number of social calls and the abundance of Lepidoptera (GLMM: Estimate = − 0.02 ± 0.01, *t* = − 1.13, *P* = 0.26). A positive relationship between the number of social vocalizations and echolocation calls was found (GLMM: Estimate = 1.75E− 3 ± 4.77E− 4, *t =* 3.67, *P* < 0.001; Fig. 3c; Table 2). Repeated analysis for each syllable type yielded similar results (Table S4, Additional file 4).

**Table 2:** Summary of optimized generalized linear mixed models

Predictors	Estimate ± s. e.	*t*	*P*	VIF	TI
Dependent variable: number of syllables					
(Intercept)	4.01 ± 0.47	8.56	< 0.001	–	–
Insect abundance	3.89E− 3 ± 1.04E− 3	3.76	< 0.001	1.13	0.88
Pielou’s evenness index	−1.22 ± 0.61	−2.01	0.048	1.47	0.68
Number of echolocation pulses	1.75E−3 ± 4.77E−4	3.67	< 0.001	1.47	0.68

The sample sizes are 30. Estimate: coefficient of the optimized model. *P* probability, *VIF* variance inflation factors, *TI* tolerance indices

### Behavioural response to playback stimuli

**Fig. 4:**
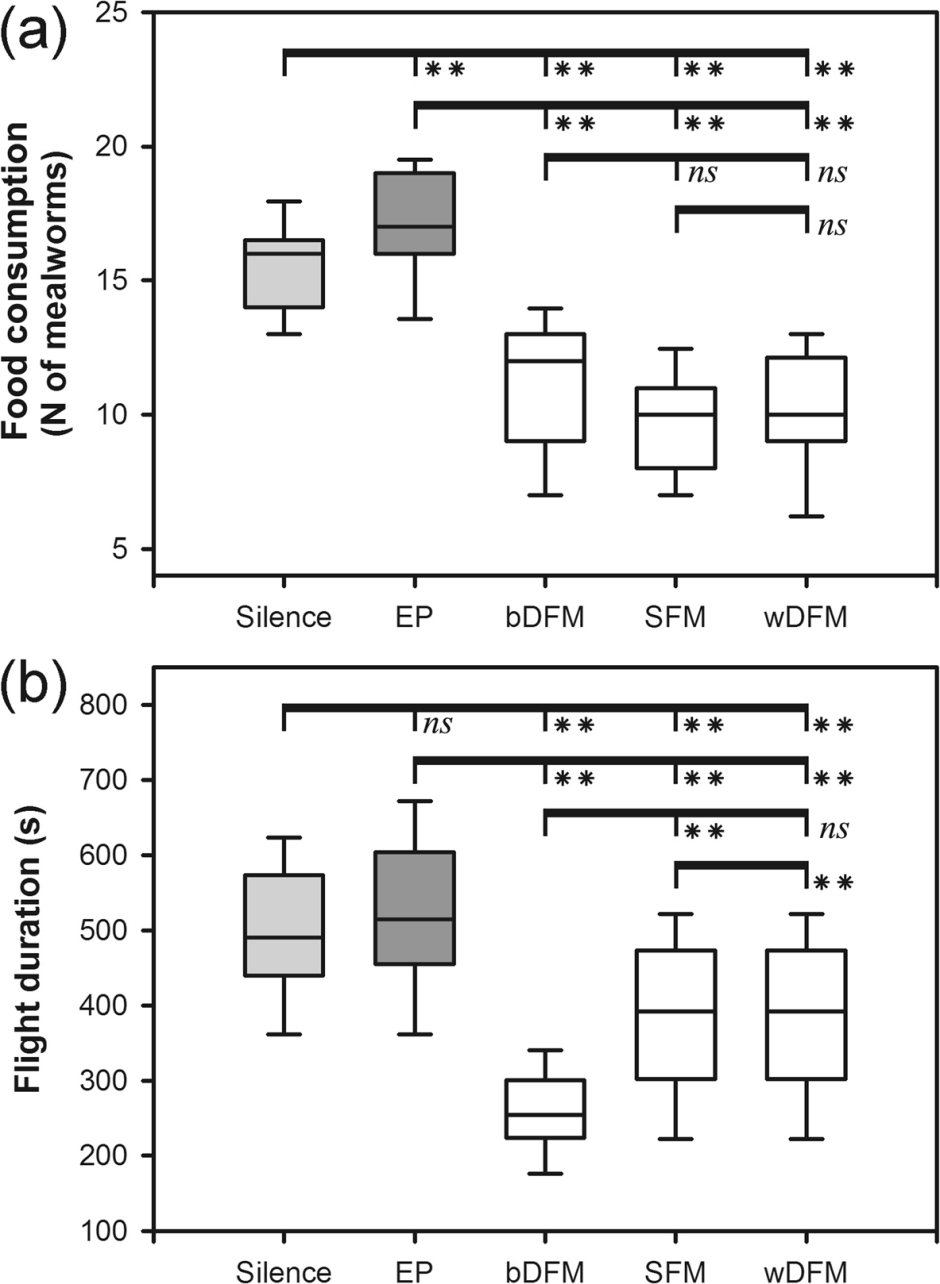
Bat foraging activity during playback experiments. **a** Food consumption. **b** Flight duration. Silence: silent control. EP: echolocation pulses. Data in box plots are the upper and lower adjacent values (highest and lowest horizontal line, respectively), 25 and 75% quartiles with median value (box). Statistical significance is based on post-hoc Tukey’s test. * *P* < 0.05. ** *P* < 0.001. ns: not significant

Foraging activity (i.e. food consumption and flight duration) of bats in the flight room was significantly lower during playback of social calls than during control trials (ANOVA: food consumption: *F*_4,165_ = 78.93, *P* < 0.001; flight duration: *F*_4,165_ = 46.26, *P* < 0.001; Fig. 4; Table S5, Additional file 5). Food consumption was dramatically reduced during playback of social calls compared to playback of echolocation pulses (Tukey’s test: bDFM: mean difference = − 6.07, *P* < 0.001; wDFM: mean difference = − 6.80, *P* < 0.001; SFM: mean difference = − 7.45, *P* < 0.001) and silence (Tukey’s test: bDFM: mean difference = − 4.36, *P* < 0.001; wDFM: mean difference = − 5.09, *P* < 0.001; SFM: mean difference = − 5.74, *P* < 0.001). The consumption of food increased when echolocation pulses were played back compared to the silent control (Tukey’s test: mean difference = 1.71, *P* = 0.007; Fig. 4a). Flight duration decreased 1.29–1.96 fold in the presence of social calls versus controls (Tukey’s test: all *P* < 0.05). Despite similar amounts of food consumption, flight duration showed significant difference when acoustically different social calls were broadcasted (Fig. 4b).

## Discussion

In this study, we investigated the function of social calls in big-footed myotis during foraging. Three lines of evidence indicate that social calls emitted by foraging big-footed myotis primarily mediate intraspecific food competition. First, the number of social vocalizations was positively related to insect abundance but was inversely related to evenness index of insects, suggesting that insect availability is an important factor underlying social calling behavior in foraging bats. Second, using echolocation pulses as a measure of feeding behavior, we found that big-footed myotis tended to reduce their foraging activities in the foraging area after the emission of bDFM, wDFM, SFM, fDFM, and dPFM-DFM calls. Third, playback of bDFM, wDFM, and SFM calls impeded individual foraging activity in the flight room, as evidenced by decreased consumption of mealworms and decreased flight duration. Consequently, these results support the food defence hypothesis that social calls can ward off food competitors.

Big-footed myotis emitted more social calls when insect diversity decreased, albeit a positive relationship existed between social vocalizations and insect abundance. The number of echolocation pulses before the production of social calls (i.e. bDFM and fDFM) was greater than these after social calls output. Playback of social calls influenced bats’ foraging attempts and food consumption. These findings indicate that social calls given by foraging big-footed myotis play a primary role in mediating intraspecific food competition. In the breeding season, food competition within bat maternal colonies is intense, owing to high energy and nutrition demands for reproductive output [30, 44, 45]. The increased group size would intensify food competition with non-kin members when pups have the ability of echolocation and active flight [46]. Vocal mediation of intraspecific food competition also occurs in some other vespertilionid bats. Pregnant northern bats (*Eptesicus nilssoni*) defend feeding territories through the production of social calls and aggressive chases [47]. Both *P. pipistrellus* and *P. pygmaeus* emit low-frequency social calls at high repetition rates in the context of feeding, leading to an apparent decrease in intraspecific foraging activity [37]. Male big brown bats utter frequency-modulated calls to claim food ownership [38], and female Asian particoloured bats employ loud screams to engage in agonistic foraging interactions [30]. However, contrary to these previous studies [37, 38, 45, 48], we found that bat social vocalizations scaled negatively with insect evenness instead of insect abundance. This suggests that competition for preferred insect prey determines the output of social calls in big-footed myotis. Indeed, further analysis of our data showed that the Pielou’s evenness index of insects was predicted remarkably well by the relative abundance of Lepidoptera (OLS: Estimate = 0.62 ± 0.15, *P* < 0.001, *R*^*2*^ = 0.42; Table S6, Additional file 6). There is also negative association between the relative abundance of Lepidoptera and total number of syllables (OLS: Estimate = − 0.57 ± 0.16, *P* = 0.001, *R*^*2*^ = 0.32; Table S6, Additional file 6). For many trawling insectivorous bats, dipteran insects are the most frequently occurring prey due to the highest abundances in aquatic habitats, whereas lepidopteran insects are the most preferred prey due to their larger size and higher energy supply [49, 50]. Taken together, we speculate that the increased competition for available food trigger the emission of social calls in foraging bats.

The emission of social calls was also positively predicted by the number of echolocation pulses from conspecifics. Since echolocation signals can be a reliable indicator of bat presence, more social calls recorded might be attributed to an increase in the number of foraging bats in the focal transect. In addition, most echolocating bats locate and identify prey by emitting echolocation pulses and receiving the echoes returning from the targets [51]. The sensory interference from neighboring conspecifics’ sonar reduces the acoustic field of view of the echolocators, largely impairing the performance of spatial orientation and foraging [52, 53]. In this circumstance, it was also possible that big-footed myotis increase the emission of social calls to chase away their potential competitors when the feeding group was dense. One recent study also discovered that the emergence of conspecifics at high densities was detrimental for prey capture, given that echolocating bats have to focus their attention on nearby conspecifics for collision avoidance [15]. Further research is invited to explore whether intraspecific sonar interference may elicit the emission of social calls in foraging bats.

Despite the consistent consumption of food, experimental bats showed differential foraging duration in response to the different syllable types. The bDFM call was most effective in inhibiting flight duration of feeding bats. The bDFM call emitted by *M. macrodactylus* is a low-frequency broadband signal with a high repetition rate, which resembles social calls used for food resource defense in pipistrelle and big brown bats [37, 38]. Low-frequency sounds transmit comparatively long distances due to frequency-dependent attenuation, and therefore facilitate social information transfer between colony members [35, 54]. Broadband calls can trigger excitatory response of neurons within bat receivers’ basolateral amygdale, the area responsible for encoding and expression of fear [55–57]. Although production of echolocation calls in flying bats incurs a small energetic cost due to the mechanical linkage between pulse emission and wingbeat, emission of social calls at greater rates may be costly for echolocating bats [58, 59]. Physiological evidence has shown that metabolic cost of lesser bulldog bats (*Noctilio albiventris*) scales positively with the rate of echolocation pulses in a communicative non-foraging context [60]. Behavioral experiments on Asian particoloured bats indicate that social call rates serve as a reliable indicator of body size, body quality, and dominance scores during agonistic interactions, suggesting that only larger and healthier individuals are capable of sustaining sound output at higher rates [30, 61]. In many insects, frogs, and birds, oxygen consumption also increases rapidly with increased call rates [62, 63]. Therefore, bDFM calls may provide reliable information about the competitive ability of the sender, intimidating potential competitors away from food sources [61]. Combined with previous research, our field recording and playback experiments confirm that bDFM calls given by foraging bats serve a food-defence function [37, 38].

Big-footed myotis tended to initiate relatively more foraging efforts during playbacks of SFM and wDFM calls compared to the bDFM signal. Our further inspection of data reveals that 4.6% of the bDFM calls were produced in the presence of feed buzzes. However, 26.7% of the SFM calls occurred in conspecifics’ terminal phases of insect pursuit, and 8.3% of the wDFM calls overlapped with feeding buzzes of conspecifics. We observed that adult females flew more than 6 min to capture the mealworms during the playback of SFM calls, ultimately resulting in a low foraging efficiency. In contrast, experimental bats foraged less than 4 min during the playback of bDFM calls. The foraging time of experimental bats was about 5 min per trial when wDFM calls were played back. Acoustically, SFM and wDFM calls exhibited remarkable overlap in frequency parameters with a conspecific’s feeding buzz I and buzz II, respectively. The duration and maximum sweep rate of frequency-modulated (FM) components in the SFM calls were nearly equivalent to those of feeding buzzes from nearby conspecifics (Figure S1, Additional file 7) [43]. Since SFM and wDFM calls overlapped temporally and spectrally with echolocation pulses and associated echoes, these calls might function in interfering with the echolocation of conspecific intruders, as documented in Brazilian free-tailed bats [39].

One of the most important benefits of group living is information transfer [64, 65]. Social animals can enhance their foraging efficiency by extracting information about food sources from conspecifics [66]. This is the case for echolocating bats, which maintain high rates of echolocation vocalizations while foraging [32]. Echolocating bats modify pulses’ temporal parameters during the search, approach, and phases of feeding buzz [51]. The inadvertent sound delivery with a specific temporal pattern provides public information on food availability, which can be perceived by conspecific bats via eavesdropping [32, 67]. Indeed, our playback of echolocation pulses during the search phase led to elevated foraging attempts and food consumption in big-footed myotis. A similar phenomenon of intraspecific acoustic eavesdropping has been found in a variety of bats, including *T. brasiliensis* [68], *Noctilio albiventris* [31], and *Rhinopoma microphyllum* [15]. Eavesdropping on food-associated echolocation calls is not confined within a species, instead observed at the interspecific level [33].

The dietary composition of the big-footed myotis was dominated by Diptera in July but was gradually replaced by Lepidoptera and Trichoptera in August. One possible cause of temporal variation in *M. macrodactylus*’ diet is the seasonal dynamics of insect diversity in the environment [69]. This is consistent with the results upon inspection of our data, as there was a tight link between prey composition in faecal pellets and insect diversity in the focal transect. Moreover, the increased energy requirements of reproduction in lactating *M. macrodactylus* might also shape the observed temporal pattern of diets, given that the majority of pups were found in late July [46]. A similar temporal variation in the diets has been previously validated in many social animals. Within the order Chiroptera, the most common insects eaten by little brown bats (*Myotis lucifugus*) vary from Diptera to Lepidoptera or Ephemeroptera over the phases of the reproductive cycle [70]. The consumption of Coleoptera by big brown bats exhibits clear seasonal and annual variation [71]. In Rickett’s big-footed bats (*Myotis pilosus*), composition of Diptera and Lepidoptera in the guano differs between summer and autumn [72].

## Conclusions

In summary, we demonstrated that the big-footed myotis utilizes multiple social calls for foraging interactions in the foraging area. They consume a diverse range of insects belonging to 59 families in 10 orders dominated by Diptera, Lepidoptera, and Trichoptera. Field survey and audio recording showed that insect abundance and diversity influenced the emission of social calls in big-footed myotis during foraging. Playbacks of social calls remarkably reduced conspecific foraging activity in the experimental site. These results provide compelling evidence supporting the hypothesis that social calls function in resource defence in wild bats. Our findings highlight the idea that competition for limited food resources correlates with the emission of social calls in foraging bats and thus yield a better understanding of the evolution of social calls in echolocating bats.

## Methods

### Survey of habitat use

During June and July 2017, we performed a field survey around the Dalazi Cave (E: 125°50′9.8″, N: 41°3′55.8″) in Yulin town, Ji’an City, Jilin, P. R. China, where a maternal colony of *M. macrodactylus* has been monitored during the past 10 years. Dozens of *M. macrodactylus* feed on aquatic insects over a seasonal river located about 500 m away from the bat roost [43]. The river ranges from 5 to 25 m in width, with some riparian vegetation and cropland along the banks. We determined foraging habitats used by *M. macrodactylus* via the line transect method based on acoustic sampling [73]. We visited the river in daylight for habitat assessment and transect planning. We chose 15 1-km transects for habitat surveys across the upstream and downstream areas of the river. The straight-line distance between the different sampling transects and bat roost ranged from 1 to 12 km.

Echolocation pulses emitted by foraging *M. macrodactylus* were recorded using an ultrasonic sound acquisition system (UltraSoundGate 116, Avisoft Bioacoustics, Berlin, Germany) connected to a laptop computer. The sampling frequency was set to 375 kHz at 16-bits/sample. A condenser microphone (UltraSoundGate CM16, Avisoft Bioacoustics, Berlin, Germany) was held 1.5 m above the riverbank by a person walking at a speed of approximately 1.5 km/h. We oriented the microphone toward the transects during acoustic sampling. We conducted acoustic recordings in each transect from 19:30 to 00:00, the time of peak feeding activity in bats. The local sunset time ranged from 19:00 to 19:12 across the recording experiments. To minimize potential effects of pseudoreplication, acoustic sampling for different transects was randomly determined prior to the onset of bat foraging. We sampled the same transect three times over separate nights without rain and strong wind. Finally, the most common transect used by feeding bats (referred to as the focal transect; Table S7, Additional file 8), as measured by the largest number of echolocation pulses and feeding buzzes, was used for further experiments.

### Insect survey and acoustic recording

To estimate the availability of food in the focal transect, we collected night-flying insects with a light trap for 30 nights during July and August in 2017 and 2018. We used a portable 100-watt mercury lamp to attract the insects. A hand net was used to capture the insects near the light trap. To reduce possible disturbance of light pollution on foraging bats, sampling of insects only lasted for 4 hours from 19:00 to 23:00 per night. The captured insects were transferred from the hand net into plastic bags and were exposed to ethyl acetate for at least 5 minutes. We sorted out all insect samples in a field station. Because some aquatic insects could not be identified to the species level according to morphology, all insect samples were assigned to at least the family level by an entomologist [74, 75]. Upon collecting the insects, another experimenter simultaneously recorded echolocation and social calls in foraging bats. Vocalizations were picked up with the ultrasonic acquisition system connected to a laptop using a sample rate of 375 kHz at 16 bits/sample. The condenser microphone was supported on a tripod 0.5 m above the ground and was positioned 6 m away from the light trap.

### Dietary composition

We employed next-generation sequencing of faecal DNA to establish the diet of *M. macrodactylus* in nine different sampling periods, to ensure that the collected insects were being consumed by these bats. We caught 72 *M. macrodactylus* (48 ♀, 24 ♂) with a mist net at the roost entrance between 23:00 and 01:00 after they had returned from nightly feeding. After identifying the sex, the trapped bats were placed separately in a clean cloth bag for faecal collection. Bats were released after faecal pellets collection, with the exception of 14 adult females that were used for later playback experiments. Males were not retained for playback experiments, given that these juveniles were harder to rear in the temporary field station. The guano pellets were placed in 2 ml Eppendorf tubes containing 95% ethanol and were individually labeled and subsequently stored at − 20 °C. We extracted DNA from 160 mg faeces per bat using the QIAamp DNA Stool Mini Kit (Qiagen). A 225 bp fragment of the cytochrome c oxidase subunit I (CO I) of insects was amplified using the primer LCO-1490 (5′-GGTCAACAAATCATAAAGATATTGG-3′) and ZBJ-ArtR2c (5′ -WACTAATCAATTWCCAAATCCTCC-3′) [76, 77]. The PCR reactions were performed following the protocol reported by Brown et al. (2013), i.e. 95 °C for 10 min, 45 cycles of 94 °C for 30 s, 52 °C for 30 s, 72 °C for 60 s, and a final extension of 72 °C for 10 min [78]. The products of three separate PCR replicates were mixed and extracted from a 2% agarose gel and further purified using the AxyPrep DNA Gel Extraction Kit (Axygen Biosciences). We quantified the final mixed PCR products using QuantiFluor-ST (Promega) according to the manufacturer’s protocol. We sequenced the products using next-generation sequencing (2 × 300 bp paired- end) on the Illumina MiSeq platform according to the standard protocols from Majorbio Bio-Pharm Technology Co. Ltd. Raw sequences were demultiplexed according to library indexes. Raw fastq files were quality-filtered and merged via Trimmomatic and FLASH [79, 80]. Molecular operational taxonomic units (MOTUs) were clustered with 97% similarity threshold using Usearch with the singletons removed and chimera filtering. To minimize the effect of sequencing errors, we removed the MOTUs that represented < 0.1% of the normalized sequences for each sample. Taxonomic identification was achieved by comparing a representative sequence of each MOTU to reference sequences in the Barcode of Life Database (BOLD; www.boldsystems.org) according to the strict and best matching criteria. The strict matching criteria is based on phylogenetic tree placement where the query sequence must be nested within a monospecific clade, whereas the best matching method assigns taxonomy based on percentage similarity [81].

### Sound processing

We analysed social calls from foraging bats in the focal transect with Avisoft-SASLab Pro version 5.2.9 (Avisoft Bioacoustics, Berlin, Germany), based on a 1024 FFT, 100% frame size, and 93.75% temporal overlap. This yielded a frequency resolution of 366 Hz and a temporal resolution of 0.1707 ms. We defined a syllable as the smallest unit of a call surrounded by periods of silence [82]. A composite was a combination of two or more simple syllabic components without an interval. A call was a sequence of two or more syllables emitted by a single bat.

**Fig. 5:**
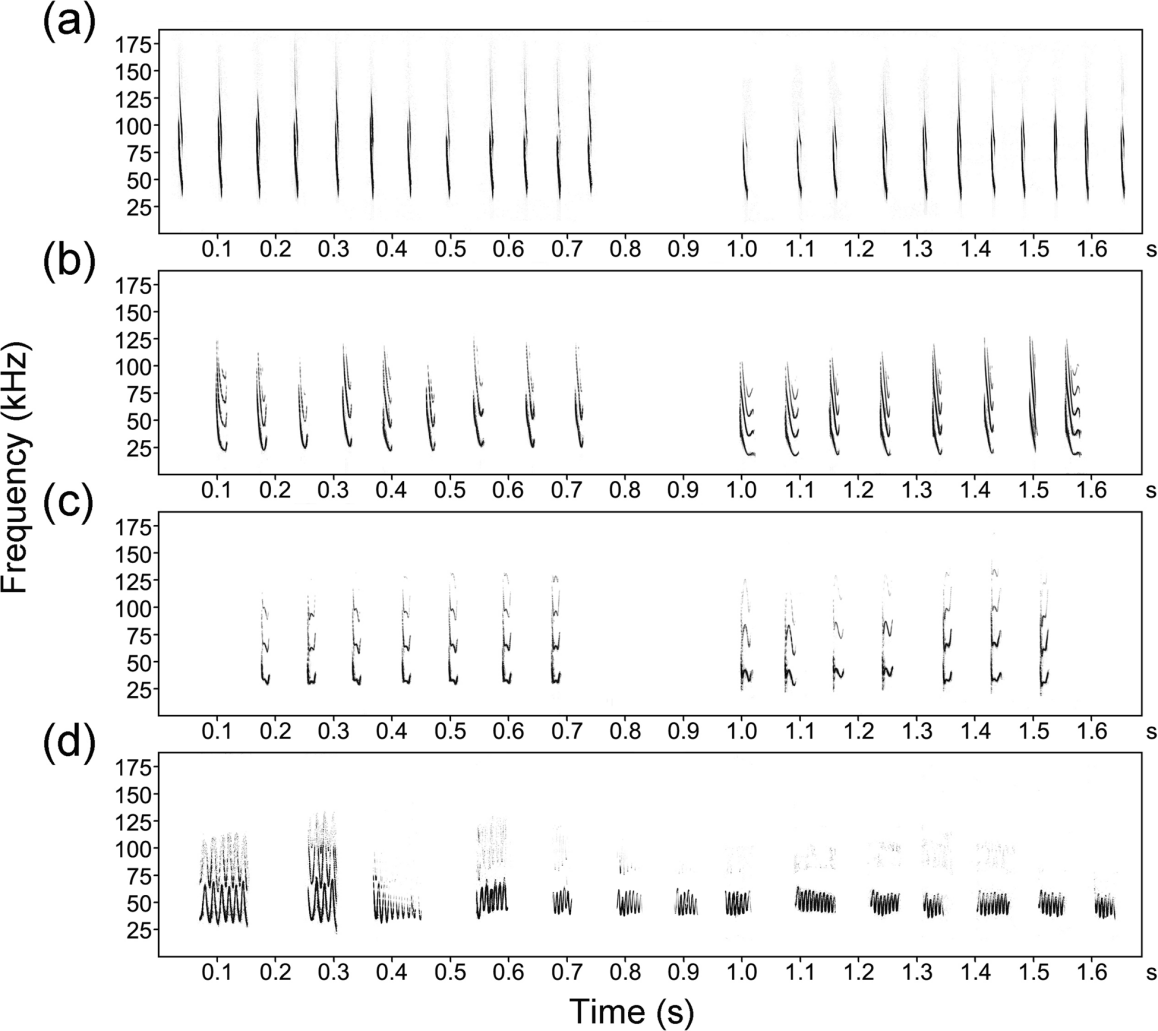
Spectrograms of echolocation and social calls used for playbacks. **a** Echolocation pulses. **b** Bent downward frequency-modulated (bDFM) call. **c** Wrinkled downward frequency-modulated (wDFM) call. **d** Sinusoidal frequency-modulated (SFM) call

We previously identified 8 syllable types in social calls from foraging *M. macrodactylus*, including bDFM, wDFM, SFM, fDFM, sDFM, CFM-DFM, dPFM-DFM and rectangular broadband noise burst (rBNB) [43]. Herein, we used three representative social calls as experimental stimuli, i.e., bDFM calls, wDFM calls, and SFM calls (Fig. 5). These calls were long monosyllabic vocalizations that differed in spectro-temporal features, and were frequently observed during intraspecific foraging interactions according our previous preliminary study [43]. The bDFM and wDFM calls had an average duration of approximately 0.6 s, consisting of 7–9 repeated syllables. SFM calls were emitted as a long monosyllabic signal that reached 0.1 s. To exclude the impact of conspecific echolocation vocalizations, we only chose social calls that were not embedded in echolocation pulse trains for playbacks. To keep the consistency in call duration, we edited each social call into a 1.7 s sound file by inserting silence segments at the end of the call using Avisoft-SASLab Pro. We did not modify the sequence of syllables to avoid the possibility of changing call syntax. We used 1.7 s silence and search-phase echolocation calls from conspecifics as two controls. The silence file was generated by Avisoft software, and echolocation calls were assembled from two original echolocation pulse sequences in search flights. Only one type of stimulus (silence segments or sounds) was played every night. Sounds were normalized to 75% of the maximum amplitude. We adjusted the sound volume of the loudspeaker so that sound intensity at 1 m was similar to the normalized value and then used a constant sound volume (6 dB) throughout the playbacks.

### Playback trials

We carried out playback experiments during September 2018. To recognize individual identity, 14 adult females were marked with numbered aluminum alloy bands (2.9 × 4.0 mm; Porzana Ltd., Winchelsea, UK) on their right forearm. These bats were maintained in a cage (1.0 × 0.8 m and 0.8 m high) in the field station, with optimum temperature (20–25 °C), humidity (50–70%), and natural light-dark cycles. During the first 2 weeks, bats were trained to feed on water and mealworms. After a two-week acclimation period, bats had free access to food in two plastic dishes positioned in the cage.

We built a temporary flight room (5 × 3 m and 2 m high) with soft polyethylene netting near the foraging habitat of the bats. The polyethylene netting was supported by five tree trunks, which prevented nocturnal insects from entering the flight room (Figure S2, Additional file 9). During each trial, 20 fresh mealworms were evenly suspended on cotton lines (0.7-mm diameter) 1.2 m above the ground in the flight room. Prior to conducting the playbacks, experimental bats were trained to capture the tethered mealworms for about 2 weeks. All bats had learned to feed on the mealworms after the training (https://datadryad.org/stash/share/712_lMrv-YdnWrytygyQLAAcmiXjFhaExQkZr57zPPg). Silence segments or sounds were presented through a loudspeaker (UltraSoundGate Player 116, Avisoft Bioacoustics, Berlin, Germany) driven by a laptop. The loudspeaker was mounted on a tripod 1.2 m above the ground at the right side of the flight room. Bats’ foraging behaviour was monitored via an infrared thermal imager (FLIR T610, FLIR System, USA) fixed on a 1.2 m tripod that was placed 5 m outside the flight room. We adjusted the angle of camera view to optimally record feeding activity of the focal bat. Playbacks commenced 30 min after local sunset. We released one bat into the flight room per trial. We randomly broadcast a playback stimulus for 15 min using loop mode when the bat began to fly and forage. Experimental bats were transferred from the flight room into the cage with cloth bags after 15 min of playbacks. We counted the number of mealworms consumed by each experimental bat, and the next trial started with new mealworms and a new bat. We aborted the trials if an individual made no foraging attempts within 5 min after release. Each bat was tested for no more than one time per night during different sound stimulation. Throughout the playback experiments, testing was repeated 2–4 times per individual under each condition depending on foraging attempts (Table S8, Additional file 10). To avoid the influence of spatial memory, we altered the locations of the mealworms in each trial (https://datadryad.org/stash/share/712_lMrv-YdnWrytygyQLAAcmiXjFhaExQkZr57zPPg).

### Data analysis

To assess bats’ dietary composition, we computed the weighted percentage of occurrence data (wPOO) and the relative read abundance data (RRA) of prey orders [83]. We employed the chi-square test to examine whether dietary composition of bats differed between sampling periods and between sexes. To quantify insect abundance, we determined the number of collected insects from each order consumed by *M. macrodactylus*. The Pielou’s evenness index (J_e_) and Shannon-Weiner index (H_e_) were applied to estimate insect diversity in different sampling periods according to the formula J_e_ = ∑^k^_i_ − P_i_ ∗ ln (P_i_)/ ln(S) and H_e_ = ∑^k^_i_ − P_i_ ∗ ln (P_i_), respectively; where S represented the total number of insect orders sampled and P_i_ represented the observed proportion of the *i*th order [84]. We counted the number of each syllable with good signal-to-noise ratios (> 45 dB) by inspecting the oscillograms and spectrograms using Avisoft software. To quantify bat foraging activities, we counted the total number of echolocation pulses louder than − 20 dB RMS. In addition, we recorded the number of echolocation pulses in the interval beginning 20 s prior to the presence of social calls and ending 20 s after social call output. The paried-samples t-tests were conducted to determine whether the presence of social calls induced a change in bat echolocation vocalizations. An ordinary least squares model (OLS) was used to examine the correlation between insect availability and composition of insects identified in the guano. We conducted a generalized linear mixed-effect model (GLMM) with quasi-Poisson family to assess the relationships among the number of social vocalizations, insect availability, and the number of echolocation pulses [85]. The abundance and Je index of insects, together with the number of echolocation pulses, were assigned as fixed variables. The H_e_ index was not included in the model because of its marked association with Je index but weak effect on the number of social vocalizations. To account for overdispersion of the model [86, 87], experimental dates (*N* = 30) were assigned as an ‘observation level’ random effect. The variance inflation factors (VIF) and tolerance indices (TI) were used to identify the collinearity between the predictor variables. We found that the VIF was less than 2 and TI was greater than 0.5, suggesting the absence of multicollinearity between the predictors (Table 1) [88]. There were no significant interactions between predictor variables based on the likelihood ratio test. We chose the optimized GLMM based on Akaike’s information criterion corrected for small sample size using the package MuMIn 1.15.6 [89].

Behavioural videos were processed with a QVOD Player 5.17 (Shenzhen Qvod Technology Co., Ltd., Guangdong, China), and blind methods were used to minimize observer bias. To quantify bat feeding activity under different conditions, we counted the number of consumed mealworms during each playback trial. Some mealworms were not fully eaten by the bats. In this case, we defined the number as one half of the food consumption. For each bat, total time spent in a flight per trial was used as another indicator of feeding activity. We conducted one-way ANOVA and post hoc Tukey’s tests to assess whether bats reduced foraging activity in the presence of experimental stimuli, given that the data satisfied the basic assumptions (i.e., random sampling, homogeneity of variances, independence of errors, and normal distribution of errors) [90]. Statistical tests were conducted two-tailed with a significance level of 0.05. Statistics were run in SPSS 24.0 (SPSS Inc., Chicago, IL, USA) and R 3.5.3. Means are given ± SE.

## Supplementary Information

**Additional file 1: Table S1.** Dietary taxa identified in *M. macrodactylus* fecal samples in different periods.

**Additional file 2: Table S2.** The average number of sampled insects in the focal transect in different periods.

**Additional file 3: Table S3.** The first five alternative generalized linear mixed models.

**Additional file 4: Table S4.** Relationships among the number of different syllables, insect availability, and number of echolocation pulses.

**Additional file 5: Table S5.** Behavioral responses display in the playback experiments.

**Additional file 6: Table S6.** Relationships among relative abundance of different insects, insect diversity, and social vocalizations.

**Additional file 7: Figure S1.** Spectrograms of echolocation pulses and sinusoidal frequency-modulated (SFM) calls. There was an overlap between the SFM type and other bats’ terminal buzzes.

**Additional file 8: Table S7.** Foraging activity in big-footed myotis across different transects.

**Additional file 9: Figure S2.** Experimental setup for playback experiments.

**Additional file 10: Table S8.** The performance of each focal bat during the different playback trials

## Data Availability

The datasets used and/or analysed during the current study are available from the corresponding author on reasonable request.

## Abbreviations

wPOO: Weighted percentage of occurrence
RRA: Relative read abundance
GLMM: Generalized linear mixed model
OLS: Ordinary least squares model
Je: Pielou’s evenness index
He: Shannon-Weiner index
AICc: Akaike information criterion corrected for small sample size
w: AICc weights
VIF: Variance inflation factors
TI: Tolerance indices
EP: Echolocation pulses
bDFM: Bent downward frequency-modulated
wDFM: Wrinkled downward frequency-modulated
SFM: Sinusoidal frequency-modulated
MOTUs: Molecular operational taxonomic units
BOLD: Barcode of life database
FFT: Fast Fourier Transform

## References

[1] Alexander RD (1974). The evolution of social behavior. Annu Rev Ecol Syst.

[2] Gil MA, Hein AM (2017). Social interactions among grazing reef fish drive material flux in a coral reef ecosystem. Proc Natl Acad Sci U S A.

[3] Silk JB, Alberts SC, Jeanne A (2003). Social bonds of female baboons enhance infant survival. Science.

[4] May D, Reboreda JC (2005). Conspecific and heterospecific social learning in shiny cowbirds. Anim Behav.

[5] Gil MA, Hein AM, Spiegel O, Baskett ML, Sih A (2018). Social information links individual behavior to population and community dynamics. Trends Ecol Evol.

[6] Chivers DP, McCormick MI, Allan BJM, Ferrari MCO (2016). Risk assessment and predator learning in a changing world: understanding the impacts of coral reef degradation. Sci Rep.

[7] Campbell LAD, Tkaczynski PJ, Lehmann J, Mouna M, Majolo B (2018). Social thermoregulation as a potential mechanism linking sociality and fitness: Barbary macaques with more social partners form larger huddles. Sci Rep.

[8] Krause J, Ruxton GD (2002). Living in groups.

[9] Yom-Tov Y (2001). An updated list and some comments on the occurrence of intraspecific nest parasitism in birds. Ibis.

[10] Torney CJ, Berdahl A, Couzin LD (2011). Signalling and the evolution of cooperative foraging in dynamic environments. PLoS Comput Biol.

[11] Galef BG, Giraldeau L-A (2001). Social influences on foraging in vertebrates: causal mechanisms and adaptive functions. Anim Behav.

[12] Yip EC, Powers KS, Aviles L (2008). Cooperative capture of large prey solves scaling challenge faced by spider societies. Proc Natl Acad Sci U S A.

[13] Le Roux A, Cherry MI, Gygax L, Manser MB (2009). Vigilance behaviour and fitness consequences: comparing a solitary foraging and an obligate group-foraging mammal. Behav Ecol Sociobiol.

[14] Baird RW, Dill LM (1996). Ecological and social determinants of group size in *transient* killer whales. Behav Ecol.

[15] Cvikel N, Berg KE, Levin E, Hurme E, Borissov I, Boonman A, Amichai E, Yovel Y (2015). Bats aggregate to improve prey search but might be impaired when their density becomes too high. Curr Biol.

[16] Clay Z, Smith CL, Blumstein DT (2012). Food-associated vocalizations in mammals and birds: what do these calls really mean?. Anim Behav.

[17] Bradbury JW, Vehrencamp SL (2011). Principles of animal communication.

[18] Judd TM, Sherman PW (1996). Naked mole-rats recruit colony mates to food sources. Anim Behav.

[19] Hauser MD, Marler P (1993). Food-associated calls in rhesus macaques (*Macaca mulatta*): I. Socioecological factors. Behav Ecol.

[20] Hauser MD, Marler P (1993). Food-associated calls in rhesus macaques (*Macaca mulatta*): II. Costs and benefits of call production and suppression. Behav Ecol.

[21] Brown CR, Brown MB, Shaffer ML (1991). Food-sharing signals among socially foraging cliff swallows. Anim Behav.

[22] Radford AN (2004). Vocal mediation of foraging competition in the cooperatively breeding green woodhoopoe (*Phoeniculus purpureus*). Behav Ecol Sociobiol.

[23] Radford AN, Ridley AR (2008). Close calling regulates spacing between foraging competitors in the group-living pied babbler. Anim Behav.

[24] Gros-Louis J (2004). The function of food-associated calls in white-faced capuchin monkeys, *Cebus capucinus*, from the perspective of the signaller. Anim Behav.

[25] Caine NG, Addington RL, Windfelder TL (1995). Factors affecting the rates of food calls given by red-bellied tamarins. Anim Behav.

[26] Kerth G (2008). Causes and consequences of sociality in bats. Bioscience.

[27] Bohn KM, Gillam EH (2017). In-flight social calls: a primer for biologists and managers studying echolocation. Can J Zool.

[28] Wu X, Pang Y, Luo B, Wang M, Feng J (2019). Function of distress calls in least horseshoe bats: a field study using playback experiments. Acta Chiropterologica.

[29] Schnitzler HU, Kalko EKV (2001). Echolocation by insect-eating bats. BioScience.

[30] Luo B, Lu GJ, Chen K, Guo DG, Huang XB, Liu Y, Feng J (2017). Social calls honestly signal female competitive ability in Asian particoloured bats. Anim Behav.

[31] Dechmann DKN, Heucke SL, Giuggioli L, Safi K, Voigt CC, Wikelski M (2009). Experimental evidence for group hunting via eavesdropping in echolocating bats. Proc R Soc B.

[32] Fenton MB (2003). Eavesdropping on the echolocation and social calls of bats. Mammal Rev.

[33] Lewanzik D, Sundaramurthy AK, Goerlitz HR (2019). Insectivorous bats integrate social information about species identity, conspecific activity and prey abundance to estimate cost–benefit ratio of interactions. J Anim Ecol.

[34] Gager Y (2019). Information transfer about food as a reason for sociality in bats. Mammal Rev.

[35] Luo JH, Koselj K, Zsebok S, Siemers BM, Goerlitz HR (2014). Global warming alters sound transmission: differential impact on the prey detection ability of echolocating bats. J R Soc Interface.

[36] Wilkinson GS, Boughman JW (1998). Social calls coordinate foraging in greater spear-nosed bats. Anim Behav.

[37] Barlow KE, Jones G (1997). Function of pipistrelle social calls: field data and a playback experiment. Anim Behav.

[38] Wright GS, Chiu C, Xian W, Wilkinson GS, Moss CF (2014). Social calls predict foraging success in big brown bats. Curr Biol.

[39] Corcoran AJ, Conner WE (2014). Bats jamming bats: food competition through sonar interference. Science.

[40] Wang L, Luo JH, Wang HN, Ou W, Jiang TL, Liu Y, Lyle D, Feng J (2014). Dynamic adjustment of echolocation pulse structure of big-footed myotis (*Myotis macrodactylus*) in response to different habitats. J Acoust Soc Am.

[41] Luo JH, Ou W, Liu Y, Wang J, Wang L, Feng J (2012). Plasticity in echolocation calls of *Myotis macrodactylus* (Chiroptera: Vespertilionidae): implications for acoustic identification. Acta Theriol.

[42] IUCN SSC (2019). The IUCN red list of threatened species.

[43] Guo D, Luo B, Zhang K, Liu M, Metzner W, Liu Y, Feng J (2019). Social vocalizations of big-footed myotis (*Myotis macrodactylus*) during foraging. Integr Zool.

[44] Belwood JJ, Fullard JH (1984). Echolocation and foraging behaviour in the Hawaiian hoary bat, *Lasiurus cinereus semotus*. Can J Zool.

[45] Racey PA, Swift SM (1985). Feeding ecology of *Pipistrellus pipistrellus* ( Chiroptera: Vespertilionidae ) during pregnancy and lactation . I . Foraging behaviour. J Anim Ecol.

[46] Liu Y, Jin LR, Metzner W, Feng J (2009). Postnatal growth and age estimation in big-footed myotis, *Myotis macrodactylus*. Acta Chiropterologica.

[47] Rydell J (1986). Feeding territoriality in female northern bats, *Eptesicus nilssoni*. Ethology.

[48] Anthony LPE, Kunz TH (1977). Feeding strategies of the little brown bat, *Myotis Lucifugus*, in southern New Hampshire. Ecology.

[49] Ward JV (1992). Aquatic insect ecology. 1. Ecology and habitat.

[50] Almenar D, Aihartza J, Goiti U, Salsamendi E, Garin I (2008). Diet and prey selection in the trawling long-fingered bat. J Zool.

[51] Schnitzler H, Kalko EK, Kunz TH, Racey PA (1998). How echolocating bats search and find food. Bats: phylogeny, morphology, echolocation, and conservation biology.

[52] Ulanovsky N, Fenton MB, Tsoar A, Korine C (2004). Dynamics of jamming avoidance in echolocating bats. Proc R Soc Lond B.

[53] Chiu C, Reddy PV, Xian W, Krishnaprasad PS, Moss CF (2010). Effects of competitive prey capture on flight behavior and sonar beam pattern in paired big brown bats, *Eptesicus fuscus*. J Exp Biol.

[54] Lawrence BD, Simmons JA (1982). Measurements of atmospheric attenuation at ultrasonic frequencies and the significance for echolocation by bats. J Acoust Soc Am.

[55] Naumann RT, Kanwal JS (2011). Basolateral amygdala responds robustly to social calls: spiking characteristics of single unit activity. J Neurophysiol.

[56] Reichert MS, Gerhardt HC (2013). Gray tree frogs, *Hyla versicolor*, give lower-frequency aggressive calls in more escalated contests. Behav Ecol Sociobiol.

[57] Medvedev A, Kanwal S (2004). Local field potentials and spiking activity in the primary auditory cortex in response to social calls. J Neurophysiol.

[58] Lancaster WC, Henson ODW, Keating AW (1995). Respiratory muscle activity in relation to vocalization in flying bats. J Exp Biol.

[59] Speakman JR, Racey PA (1991). No cost of echolocation for bats in flight. Nature.

[60] Dechmann D, Wikelski M, Noordwijk H, Voigt C, Voigt-Heucke S (2013). Metabolic costs of bat echolocation in a non-foraging context support a role in communication. Front Physiol.

[61] Zhao X, Jiang TL, Gu H, Liu H, Sun CN, Liu Y, Feng J (2018). Are aggressive vocalizations the honest signals of body size and quality in female Asian particoloured bats?. Behav Ecol Sociobiol.

[62] Bailey W, Withers P, Endersby M (1993). The energetic costs of calling in the bushcricket Requena verticalis (Orthoptera: Tettigoniidae: Listroscelidinae). J Exp Biol.

[63] Taigen TL, Wells KD, Marsh RL (1985). The enzymatic basis of high metabolic rates in calling frogs. Physiol Zool.

[64] Beauchamp G, Belisle M, Giraldeau LA (1997). Influence of conspecific attraction on the spatial distribution of learning foragers in a patchy habitat. J Anim Ecol.

[65] Valone TJ, Templeton JJ (2002). Public information for the assessment of quality: a widespread social phenomenon. Philos Trans R Soc Lond.

[66] Safi K, Kerth G (2007). Comparative analyses suggest that information transfer promoted sociality in male bats in the temperate zone. Am Nat.

[67] Balcombe JP, Fenton MB (1988). Eavesdropping by bats: the influence of echolocation call design and foraging strategy. Ethology.

[68] Gillam EH (2007). Eavesdropping by bats on the feeding buzzes of conspecifics. Can J Zool.

[69] Peng RK, Sutton SL, Fletcher CR (1992). Spatial and temporal distribution patterns of flying Diptera. Proc Zool Soc London.

[70] Clare EL, Barber BR, Sweeney BW, Hebert PD, Fenton MB (2011). Eating local: influences of habitat on the diet of little brown bats (*Myotis lucifugus*). Mol Ecol.

[71] Clare EL, Symondson WO, Fenton MB (2014). An inordinate fondness for beetles? Variation in seasonal dietary preferences of night-roosting big brown bats (*Eptesicus fuscus*). Mol Ecol.

[72] Chang Y, Song S, Li A, Zhang Y, Li Z, Xiao Y, Jiang T, Feng J, Lin A (2019). The roles of morphological traits, resource variation and resource partitioning associated with the dietary niche expansion in the fish-eating bat *Myotis pilosus*. Mol Ecol.

[73] Vaughan N, Jones G, Harris S (1997). Habitat use by bats (Chiroptera) assessed by means of a broad-band acoustic method. J Appl Ecol.

[74] Li JH, Yang F, Wang Q, Pan HS, Yuan HB, Lu YH (2017). Predation by generalist arthropod predators on *Apolygus lucorum* (Hemiptera: Miridae): molecular gut-content analysis and field-cage assessment. Pest Manag Sci.

[75] Yuan HB, Li JH, Liu YQ, Cui L, Lu YH, Xu XY, Li Z, Wu KM, Desneux N (2017). Lethal, sublethal and transgenerational effects of the novel chiral neonicotinoid pesticide cycloxaprid on demographic and behavioral traits of *Aphis gossypii* (Hemiptera: Aphididae). Insect Sci.

[76] Folmer O, Black M, Wr H, Lutz R, Vrijenhoek R (1994). DNA primers for amplification of mitochondrial cytochrome C oxidase subunit I from diverse metazoan invertebrates. Mol Mar Biol Biotechnol.

[77] Zeale MR, Butlin RK, Barker GL, Lees DC, Jones G (2011). Taxon-specific PCR for DNA barcoding arthropod prey in bat faeces. Mol Ecol Resour.

[78] Brown D, Burger R, Cole N, Vencatasamy AD, Clare E, Montazam A, Symondson WOC (2013). Dietary competition between the alien Asian musk shrew (*Suncus murinus*) and a re-introduced population of Telfair’s skink (*Leiolopisma telfairii*). Mol Ecol.

[79] Bolger AM, Lohse M, Usadel B (2014). Trimmomatic: a flexible trimmer for Illumina sequence data. Bininformatics.

[80] Magoč T, Salzberg SL (2011). FLASH: fast length adjustment of short reads to improve genome assemblies. Bioinformatics.

[81] Razgour O, Clare EL, Zeale MR, Hanmer J, Schnell IB, Rasmussen M, Gilbert TP, Jones G (2011). High-throughput sequencing offers insight into mechanisms of resource partitioning in cryptic bat species. Ecol Evol.

[82] Kanwal JS, Matsumura S, Ohlemiller K, Suga N (1994). Analysis of acoustic elements and syntax in communication sounds emitted by mustached bats. J Acoust Soc Am.

[83] Deagle BE, Thomas AC, McInnes JC, Clarke LJ, Vesterinen EJ, Clare EL, Kartzinel TR, Eveson JP (2019). Counting with DNA in metabarcoding studies: how should we convert sequence reads to dietary data?. Mol Ecol.

[84] Begon M, Harper JL, Townsend CR (1986). Ecology: individuals, populations and communities.

[85] Crawley MJ (2013). The R book.

[86] O’Hara RB, Kotze DJ (2010). Do not log-transform count data. Methods Ecol Evol.

[87] Harrison XA (2014). Using observation-level random effects to model overdispersion in count data in ecology and evolution. PeerJ.

[88] Marcoulides KM, Raykov T (2019). Evaluation of variance inflation factors in regression models using latent variable modeling methods. Educ Psychol Meas.

[89] Barton K. Package ‘MuMIn’. 2016 p. 18. Available at https://cran.r-project.org/web/packages/MuMIn/index.html.

[90] Hesamian G (2016). One-way ANOVA based on interval information. Int J Syst Sci.

