# Supplementary material for: Social calls influence the foraging behavior in wild big-footed myotis
Source: Front Zool. 2021 Jan 7;18:3. doi: 10.1186/s12983-020-00384-8 (PMC7791762; doi:10.1186/s12983-020-00384-8)
Supplement: Supplementary file 1 — Additional file 1: Table S1. Dietary taxa identified in M. macrodactylus fecal samples in different periods. [file 12983_2020_384_MOESM1_ESM.docx]

**Table S1**

Dietary taxa identified in *M. macrodactylus* fecal samples in different periods

|  | 2017 | | | | | 2018 | | | |
| --- | --- | --- | --- | --- | --- | --- | --- | --- | --- |
|  | July | | | August | | July | August | | |
| **wPOO (%)** | Early | Middle | Late | Early | Middle | Late | Early | Middle | Late |
| Diptera | 45.83 | 25.00 | 33.33 | 27.33 | 26.96 | 18.80 | 26.67 | 19.30 | 27.21 |
| Chironomidae | 20.83 | 7.14 | 12.50 | 56.67 | 23.15 | 7.64 | 10.0 | 15.36 | 25.00 |
| Limoniidae | 0.00 | 9.52 | 0.00 | 5.00 | 2.78 | 10.42 | 2.90 | 9.76 | 0.00 |
| Culicidae | 0.00 | 0.00 | 8.33 | 0.00 | 16.67 | 0.00 | 0.0 | 2.98 | 6.48 |
| Tipulidae | 0.00 | 0.00 | 0.00 | 0.00 | 5.56 | 0.00 | 2.90 | 0.00 | 0.00 |
| Hippoboscidae | 0.00 | 4.76 | 8.33 | 5.00 | 0.00 | 0.00 | 0.00 | 12.14 | 7.41 |
| Psychodidae | 8.33 | 2.38 | 0.00 | 5.00 | 0.00 | 4.86 | 0.00 | 1.43 | 9.26 |
| Stratiomyidae | 0.00 | 0.00 | 0.00 | 0.00 | 0.00 | 2.78 | 0.00 | 0.00 | 2.78 |
| Muscidae | 0.00 | 0.00 | 0.00 | 0.00 | 0.00 | 0.00 | 0.00 | 1.43 | 0.00 |
| Drosophilidae | 0.00 | 2.38 | 0.00 | 6.67 | 6.48 | 0.00 | 2.90 | 7.98 | 0.00 |
| Dolichopodidae | 0.00 | 0.00 | 0.00 | 0.00 | 0.00 | 0.00 | 7.10 | 0.00 | 0.00 |
| Calliphoridae | 0.00 | 0.00 | 0.00 | 0.00 | 0.00 | 4.17 | 0.00 | 1.79 | 0.00 |
| Phoridae | 0.00 | 0.00 | 0.00 | 0.00 | 2.78 | 0.00 | 0.00 | 0.00 | 0.00 |
| Cecidomyiidae | 0.00 | 2.38 | 0.00 | 5.00 | 0.00 | 0.00 | 0.00 | 0.00 | 0.00 |
| Ephydridae | 0.00 | 0.00 | 0.00 | 0.00 | 0.00 | 0.00 | 0.00 | 1.79 | 0.00 |
| Sciaridae | 0.00 | 0.00 | 0.00 | 0.00 | 0.00 | 0.00 | 0.00 | 1.43 | 0.00 |
| Unidentified | 70.83 | 71.43 | 70.83 | 16.67 | 42.59 | 70.14 | 74.30 | 43.93 | 49.07 |
| Trichoptera | 0.00 | 25.00 | 12.50 | 27.33 | 17.70 | 18.80 | 21.90 | 19.30 | 21.15 |
| Lepidoptera | 45.83 | 25.00 | 33.33 | 27.33 | 23.25 | 18.80 | 26.67 | 19.30 | 20.84 |
| Ephemeroptera | 0.00 | 15.48 | 6.25 | 5.00 | 3.44 | 8.03 | 5.95 | 9.30 | 3.33 |
| Hemiptera | 0.00 | 4.76 | 14.58 | 4.00 | 9.37 | 7.55 | 5.24 | 9.74 | 2.81 |
| Hymenoptera | 0.00 | 2.38 | 0.00 | 0.00 | 15.85 | 6.16 | 2.86 | 2.38 | 3.12 |
| Coleoptera | 8.33 | 2.38 | 0.00 | 9.00 | 1.85 | 18.80 | 10.71 | 15.46 | 10.00 |
| Orthoptera | 0.00 | 0.00 | 0.00 | 0.00 | 1.59 | 2.12 | 0.00 | 2.56 | 10.24 |
| Odonata | 0.00 | 0.00 | 0.00 | 0.00 | 0.00 | 0.93 | 0.00 | 1.10 | 0.00 |
| Neuroptera | 0.00 | 0.00 | 0.00 | 0.00 | 0.00 | 0.00 | 0.00 | 1.54 | 1.30 |
| **RRA (%)** |  |  |  |  |  |  |  |  |  |
| Diptera | 86.26 | 57.83 | 68.86 | 14.74 | 39.61 | 62.74 | 53.47 | 60.03 | 31.14 |
| Chironomidae | 0.33 | 0.39 | 0.26 | 29.78 | 1.63 | 2.20 | 3.35 | 23.29 | 3.72 |
| Limoniidae | 0.00 | 5.47 | 0.00 | 3.73 | 0.11 | 0.40 | 0.15 | 13.89 | 0.00 |
| Culicidae | 0.00 | 0.00 | 2.29 | 0.00 | 4.52 | 0.00 | 0.00 | 0.49 | 0.13 |
| Tipulidae | 0.00 | 0.00 | 0.00 | 0.00 | 0.54 | 0.00 | 0.06 | 0.00 | 0.00 |
| Hippoboscidae | 0.00 | 0.32 | 0.82 | 0.42 | 0.00 | 0.00 | 0.00 | 1.38 | 4.09 |
| Psychodidae | 0.21 | 0.08 | 0.00 | 0.82 | 0.00 | 0.28 | 0.00 | 0.03 | 1.93 |
| Stratiomyidae | 0.00 | 0.00 | 0.00 | 0.00 | 0.00 | 6.14 | 0.00 | 0.00 | 0.05 |
| Muscidae | 0.00 | 0.00 | 0.00 | 0.00 | 0.00 | 0.00 | 0.00 | 0.03 | 0.00 |
| Drosophilidae | 0.00 | 0.05 | 0.00 | 0.21 | 0.10 | 0.00 | 0.11 | 0.99 | 0.00 |
| Dolichopodidae | 0.00 | 0.00 | 0.00 | 0.00 | 0.00 | 0.00 | 0.38 | 0.00 | 0.00 |
| Calliphoridae | 0.00 | 0.00 | 0.00 | 0.00 | 0.00 | 0.02 | 0.00 | 2.02 | 0.00 |
| Phoridae | 0.00 | 0.00 | 0.00 | 0.00 | 1.05 | 0.00 | 0.00 | 0.00 | 0.00 |
| Cecidomyiidae | 0.00 | 2.33 | 0.00 | 3.09 | 0.00 | 0.00 | 0.00 | 0.00 | 0.00 |
| Ephydridae | 0.00 | 0.00 | 0.00 | 0.00 | 0.00 | 0.00 | 0.00 | 0.56 | 0.00 |
| Sciaridae | 0.00 | 0.00 | 0.00 | 0.00 | 0.00 | 0.00 | 0.00 | 0.07 | 0.00 |
| Unidentified | 99.46 | 91.37 | 96.63 | 61.96 | 92.04 | 90.96 | 95.94 | 57.24 | 90.08 |
| Trichoptera | 0.00 | 13.30 | 22.22 | 41.75 | 8.44 | 5.89 | 25.40 | 18.51 | 46.46 |
| Lepidoptera | 12.76 | 23.63 | 1.40 | 42.55 | 33.17 | 20.03 | 12.87 | 15.04 | 18.83 |
| Ephemeroptera | 0.00 | 1.25 | 7.49 | 0.01 | 0.11 | 0.26 | 0.63 | 0.10 | 0.09 |
| Hemiptera | 0.00 | 1.00 | 0.03 | 0.05 | 6.32 | 0.19 | 0.50 | 0.07 | 0.34 |
| Hymenoptera | 0.00 | 0.12 | 0.00 | 0.00 | 7.24 | 0.00 | 0.88 | 0.01 | 0.01 |
| Coleoptera | 0.98 | 2.87 | 0.00 | 0.89 | 0.00 | 10.81 | 6.26 | 1.75 | 0.82 |
| Orthoptera | 0.00 | 0.00 | 0.00 | 0.00 | 5.10 | 0.07 | 0.00 | 4.37 | 1.78 |
| Odonata | 0.00 | 0.00 | 0.00 | 0.00 | 0.00 | 0.00 | 0.00 | 0.05 | 0.00 |
| Neuroptera | 0.00 | 0.00 | 0.00 | 0.00 | 0.00 | 0.00 | 0.00 | 0.07 | 0.53 |

wPOO: weighted percentage of occurrence. RRA: relative read abundance.
