# Supplementary material for: Social calls influence the foraging behavior in wild big-footed myotis
Source: Front Zool. 2021 Jan 7;18:3. doi: 10.1186/s12983-020-00384-8 (PMC7791762; doi:10.1186/s12983-020-00384-8)
Supplement: Supplementary file 2 — Additional file 2: Table S2. The average number of sampled insects in the focal transect in different periods. [file 12983_2020_384_MOESM2_ESM.docx]

**Table S2**

The average number of sampled insects in the focal transect in different periods

| Insect  availability | 2017 | | | | | 2018 | | | |
| --- | --- | --- | --- | --- | --- | --- | --- | --- | --- |
|  | July | | | August | | July | August | | |
|  | Early | Middle | Late | Early | Middle | Late | Early | Middle | Late |
| Diptera | 64.00 | 136.67 | 109.00 | 8.50 | 47.00 | 111.50 | 123.75 | 94.75 | 16.50 |
| Chironomidae | 35.67 | 67.67 | 60.00 | 2.25 | 26.33 | 54.75 | 89.75 | 63.00 | 6.50 |
| Limoniidae | 4.00 | 27.00 | 12.33 | 1.75 | 3.00 | 4.50 | 11.50 | 7.50 | 2.00 |
| Culicidae | 4.33 | 9.33 | 7.00 | 0.75 | 7.00 | 1.00 | 3.25 | 4.25 | 4.00 |
| Simuliidae | 5.33 | 16.00 | 20.00 | 0.25 | 6.33 | 38.50 | 0.50 | 3.25 | 1.00 |
| Tipulidae | 0.33 | 0.33 | 0.33 | 0.00 | 1.67 | 1.50 | 0.75 | 0.75 | 1.00 |
| Hippoboscidae | 3.67 | 1.67 | 0.33 | 0.75 | 0.33 | 0.25 | 2.50 | 2.00 | 0.00 |
| Psychodidae | 1.67 | 2.33 | 1.00 | 0.25 | 0.67 | 0.25 | 2.00 | 0.25 | 0.00 |
| Stratiomyidae | 0.33 | 1.00 | 0.00 | 0.50 | 0.67 | 0.00 | 1.00 | 3.25 | 0.50 |
| Muscidae | 2.33 | 1.67 | 0.00 | 0.00 | 0.33 | 0.25 | 1.50 | 1.00 | 0.50 |
| Drosophilidae | 0.67 | 0.00 | 0.00 | 0.00 | 0.33 | 0.50 | 0.75 | 0.75 | 0.00 |
| Dolichopodidae | 0.67 | 0.67 | 0.00 | 0.00 | 0.00 | 0.75 | 1.25 | 0.75 | 0.00 |
| Calliphoridae | 0.00 | 0.00 | 0.33 | 0.00 | 0.00 | 0.00 | 0.25 | 0.50 | 0.00 |
| Sarcophagidae | 0.33 | 0.67 | 0.33 | 0.25 | 0.00 | 0.50 | 1.25 | 0.00 | 0.00 |
| Syrphidae | 1.00 | 0.33 | 0.00 | 0.00 | 0.00 | 0.75 | 0.00 | 0.25 | 0.00 |
| Phoridae | 0.67 | 0.00 | 0.00 | 0.00 | 0.00 | 0.50 | 0.00 | 0.00 | 0.00 |
| Tephritidae | 0.00 | 0.00 | 0.00 | 0.00 | 0.00 | 0.25 | 0.00 | 0.50 | 0.00 |
| Unidentified | 3.00 | 8.00 | 7.33 | 1.75 | 0.33 | 7.25 | 7.50 | 6.75 | 1.00 |
| Trichoptera | 9.00 | 10.00 | 1.00 | 1.00 | 3.00 | 13.50 | 41.00 | 19.50 | 4.00 |
| Stenopsychiade | 0.33 | 2.33 | 0.33 | 0.00 | 0.67 | 0.50 | 1.25 | 1.75 | 1.00 |
| Hydropsychidae | 2.33 | 3.00 | 0.00 | 0.25 | 0.67 | 3.00 | 11.25 | 5.75 | 1.00 |
| Goeridae | 0.00 | 0.33 | 0.00 | 0.00 | 0.00 | 0.25 | 1.75 | 0.75 | 0.00 |
| Phryganeidae | 0.33 | 0.00 | 0.00 | 0.00 | 0.00 | 0.00 | 1.50 | 1.00 | 0.00 |
| Lepidostomatidae | 0.00 | 0.00 | 0.00 | 0.00 | 0.00 | 0.00 | 0.25 | 0.25 | 0.00 |
| Philopotamidae | 2.67 | 1.33 | 0.33 | 0.50 | 0.67 | 3.25 | 7.00 | 3.75 | 1.50 |
| Odontoceridae | 0.33 | 0.00 | 0.00 | 0.00 | 0.00 | 0.75 | 3.00 | 2.50 | 0.00 |
| Leptoceridae | 1.33 | 1.67 | 0.00 | 0.25 | 0.67 | 3.75 | 11.75 | 2.00 | 0.50 |
| Unidentified | 1.67 | 1.33 | 0.33 | 0.00 | 0.33 | 2.00 | 3.25 | 1.75 | 0.00 |
| Lepidoptera | 21.67 | 16.67 | 4.67 | 3.00 | 4.67 | 5.75 | 10.25 | 9.25 | 32.00 |
| Lithosiidae | 6.33 | 2.67 | 0.00 | 0.25 | 0.33 | 0.00 | 0.00 | 0.75 | 8.00 |
| Pyralidae | 7.00 | 6.33 | 1.00 | 0.25 | 1.67 | 2.75 | 3.75 | 1.25 | 9.50 |
| Sphingidae | 2.33 | 0.00 | 0.00 | 0.00 | 0.00 | 0.00 | 0.25 | 0.00 | 0.00 |
| Lymantriidae | 0.33 | 3.33 | 0.00 | 1.00 | 0.00 | 0.00 | 0.25 | 0.25 | 0.50 |
| Limacodidae | 1.33 | 0.00 | 0.33 | 0.00 | 0.00 | 0.00 | 0.00 | 0.00 | 0.00 |
| Geometridae | 0.33 | 0.67 | 0.33 | 0.00 | 0.00 | 0.75 | 0.25 | 0.50 | 1.50 |
| Arctiidae | 0.33 | 0.33 | 1.00 | 0.00 | 0.00 | 0.25 | 0.25 | 0.00 | 0.00 |
| Bombycidae | 0.00 | 0.67 | 0.33 | 0.00 | 0.00 | 0.00 | 0.25 | 0.00 | 0.00 |
| Noctuidae | 0.00 | 0.33 | 0.00 | 0.25 | 0.33 | 1.25 | 0.50 | 1.75 | 1.50 |
| Lasiocampidae | 0.00 | 0.00 | 0.67 | 0.00 | 0.00 | 0.00 | 0.00 | 0.25 | 0.00 |
| Notodontidae | 0.00 | 0.00 | 0.33 | 0.00 | 0.00 | 0.25 | 0.00 | 0.00 | 0.00 |
| Tortricidae | 0.00 | 0.00 | 0.00 | 0.25 | 0.33 | 0.00 | 0.00 | 0.00 | 0.00 |
| Drepanidae | 0.00 | 0.00 | 0.00 | 0.00 | 0.33 | 0.00 | 0.00 | 0.00 | 0.00 |
| Crambidae | 0.00 | 0.00 | 0.00 | 0.00 | 0.00 | 0.25 | 0.00 | 0.25 | 0.00 |
| Saturniidae | 0.00 | 0.00 | 0.00 | 0.00 | 0.00 | 0.00 | 0.25 | 0.00 | 0.00 |
| Thyatiridae | 0.00 | 0.00 | 0.00 | 0.00 | 0.00 | 0.00 | 0.25 | 0.00 | 0.00 |
| Unidentified | 3.67 | 2.33 | 0.67 | 1.00 | 1.67 | 0.25 | 4.25 | 4.25 | 11.00 |
| Ephemeroptera | 0.00 | 15.67 | 3.67 | 30.75 | 89.33 | 54.75 | 87.25 | 92.50 | 54.00 |
| Ephemeridae | 0.00 | 1.67 | 0.67 | 5.25 | 5.00 | 7.75 | 17.50 | 5.25 | 0.00 |
| Heptageniidae | 0.00 | 3.33 | 0.67 | 13.50 | 26.33 | 3.00 | 36.75 | 27.00 | 2.00 |
| Baetidae | 0.00 | 4.00 | 1.33 | 2.00 | 21.00 | 25.75 | 13.25 | 20.75 | 36.00 |
| Potamanthidae | 0.00 | 0.33 | 0.00 | 4.50 | 19.67 | 8.25 | 1.25 | 19.50 | 0.00 |
| Isonychiidae | 0.00 | 3.00 | 1.00 | 1.25 | 2.67 | 0.75 | 1.25 | 1.25 | 0.50 |
| Unidentified | 0.00 | 3.33 | 0.00 | 4.25 | 14.67 | 9.25 | 17.25 | 18.75 | 15.50 |
| Hemiptera | 0.00 | 0.67 | 0.00 | 0.25 | 0.67 | 0.25 | 0.50 | 1.00 | 0.00 |
| Hymenoptera | 0.67 | 0.67 | 0.00 | 0.25 | 1.00 | 0.00 | 1.00 | 2.50 | 0.50 |
| Coleoptera | 2.00 | 1.33 | 0.00 | 0.00 | 1.00 | 1.00 | 1.00 | 1.25 | 0.00 |
| Orthoptera | 4.00 | 4.67 | 7.67 | 0.00 | 0.67 | 0.25 | 0.00 | 0.25 | 1.50 |
| Neuroptera | 2.33 | 0.33 | 0.00 | 0.25 | 0.00 | 0.00 | 1.50 | 0.00 | 0.50 |
