# Supplementary material for: Social calls influence the foraging behavior in wild big-footed myotis
Source: Front Zool. 2021 Jan 7;18:3. doi: 10.1186/s12983-020-00384-8 (PMC7791762; doi:10.1186/s12983-020-00384-8)
Supplement: Supplementary file 3 — Additional file 3: Table S3. The first five alternative generalized linear mixed models. [file 12983_2020_384_MOESM3_ESM.docx]

**Table S3**

The first five alternative generalized linear mixed models

| Dependent variable | Predictors | Intercept | AICc | *w* |
| --- | --- | --- | --- | --- |
| Number of syllables | **Insect + Je + Echolocation** | **4.01** | **318.1** | **0.556** |
|  | Insect + Echolocation | 3.14 | 318.6 | 0.422 |
|  | Insect + Je | 4.94 | 326.0 | 0.011 |
|  | Je + Echolocation | 4.80 | 326.7 | 0.008 |
|  | Echolocation | 3.69 | 328.4 | 0.003 |

The sample sizes are 30. Models are ranked according to their AICc values from the best to the worst. Insect: Insect abundance. Je: Pielou’s evenness index. Echolocation: number of echolocation pulses. AICc: Akaike information criterion corrected for small sample size. *w*: AICc weights. Data in bold represent the optimized linear model.
