# Supplementary material for: Social calls influence the foraging behavior in wild big-footed myotis
Source: Front Zool. 2021 Jan 7;18:3. doi: 10.1186/s12983-020-00384-8 (PMC7791762; doi:10.1186/s12983-020-00384-8)
Supplement: Supplementary file 4 — Additional file 4: Table S4. Relationships among the number of different syllables, insect availability, and number of echolocation pulses. [file 12983_2020_384_MOESM4_ESM.docx]

**Table S4**

Relationships among the number of different syllables, insect availability, and number of echolocation pulses

| Syllables | Predictors | Estimate ± s. e. | *t* | *P* |
| --- | --- | --- | --- | --- |
| bDFM | Insect abundance | **6.1E−3 ± 1.6E−3** | **3.79** | **< 0.001** |
|  | Pielou’s evenness index | **−2.02 ± 0.97** | **−2.08** | **0.038** |
|  | Number of EP | **2.2E−3 ± 7.4E−4** | **2.97** | **0.003** |
| sDFM | Insect abundance | 4.2E−3 ± 2.5E−3 | 1.69 | 0.091 |
|  | Pielou’s evenness index | **−3.02 ± 1.47** | **−2.06** | **0.040** |
|  | Number of EP | 2.2E−3 ± 1.1E−3 | 1.93 | 0.053 |
| fDFM | Insect abundance | **4.6E−3 ± 1.8E−3** | **2.53** | **0.012** |
|  | Pielou’s evenness index | 2.15 ± 1.16 | 1.86 | 0.063 |
|  | Number of EP | **2.4E−3 ± 8.3E−4** | **2.91** | **0.004** |
| wDFM | Insect abundance | 1.2E−3 ± 1.8E−3 | 0.66 | 0.509 |
|  | Pielou’s evenness index | −1.27 ± 1.05 | −1.21 | 0.225 |
|  | Number of EP | **1.9E−3 ± 8.1E−4** | **2.37** | **0.018** |
| SFM | Insect abundance | **6.3E−3 ± 2.3E−3** | **2.67** | **0.008** |
|  | Pielou’s evenness index | **3.89 ± 1.63** | **2.39** | **0.017** |
|  | Number of EP | 1.8E−3 ± 1.1E−3 | 1.65 | 0.099 |
| CFM-DFM | Insect abundance | 3.1E−3 ± 2.1E−3 | 1.47 | 0.141 |
|  | Pielou’s evenness index | 0.73 ± 1.31 | 0.56 | 0.576 |
|  | Number of EP | **2.4E−3 ± 1.0E−3** | **2.40** | **0.016** |
| dPFM-DFM | Insect abundance | **9.0E−3 ± 2.7E−3** | **3.34** | **0.001** |
|  | Pielou’s evenness index | 0.89 ± 1.63 | 0.55 | 0.583 |
|  | Number of EP | 1.9E−3 ± 1.3E−3 | 1.54 | 0.123 |

The sample sizes are 30. bDFM: bent downward frequency modulation. sDFM: steep downward frequency modulation. fDFM: flattened downward frequency modulation. wDFM: wrinkled downward frequency modulation. SFM: sinusoidal frequency modulation. CFM-DFM: chevron frequency modulation-downward frequency modulation. dPFM-DFM: downward paraboloid frequency modulation-downward frequency modulation. EP: echolocation pulses.
