# Supplementary material for: Social calls influence the foraging behavior in wild big-footed myotis
Source: Front Zool. 2021 Jan 7;18:3. doi: 10.1186/s12983-020-00384-8 (PMC7791762; doi:10.1186/s12983-020-00384-8)
Supplement: Supplementary file 5 — Additional file 5: Table S5. Behavioral responses display in the playback experiments. [file 12983_2020_384_MOESM5_ESM.docx]

**Table S5**

Behavioral responses display in the playback experiments

| Types of signals | Number of trials | Food consumption | Flight duration |
| --- | --- | --- | --- |
| Silence | 50 | 15.36 ± 1.80 | 494.98 ± 97.23 |
| EP | 30 | 17.07 ± 2.16 | 514.00 ± 109.37 |
| bDFM | 30 | 11.00 ± 2.50 | 262.57 ± 71.37 |
| SFM | 30 | 9.62 ± 2.10 | 384.10 ± 115.31 |
| wDFM | 30 | 10.27 ± 2.37 | 300.70 ± 78.89 |

Values are given as mean ± SD. Silence: silence control. EP: echolocation pulses. Food consumption: the number of consumed provisioned mealworms; Flight duration: time spent in flight (seconds).
