# Supplementary material for: Social calls influence the foraging behavior in wild big-footed myotis
Source: Front Zool. 2021 Jan 7;18:3. doi: 10.1186/s12983-020-00384-8 (PMC7791762; doi:10.1186/s12983-020-00384-8)
Supplement: Supplementary file 6 — Additional file 6: Table S6. Relationships among relative abundance of different insects, insect diversity, and social vocalizations. [file 12983_2020_384_MOESM6_ESM.docx]

**Table S6**

Relationships among relative abundance of different insects, insect diversity, and number of social vocalizations

| Response variable | Predictors | Estimate ± s. e. | *P* | *R^2^* |
| --- | --- | --- | --- | --- |
| Pielou’s evenness  index | Lepidoptera | **0.62 ± 0.15** | **<0.001** | **0.42** |
|  | Diptera | −0.30 ± 0.18 | 0.107 | 0.09 |
|  | Trichoptera | 0.21 ± 0.19 | 0.270 | 0.04 |
|  | Ephemeroptera | −0.08 ± 0.19 | 0.684 | 0.01 |
|  | Neuroptera | **0.37 ± 0.18** | **0.043** | **0.14** |
|  | Coleoptera | 0.13 ± 0.19 | 0.506 | 0.02 |
|  | Hymenoptera | 0.33 ± 0.18 | 0.080 | 0.11 |
|  | Hemiptera | 0.34 ± 0.18 | 0.068 | 0.11 |
|  | Orthoptera | −0.11 ± 0.19 | 0.579 | 0.01 |
| Number of  all syllables | Lepidoptera | **−0.57 ± 0.16** | **0.001** | **0.32** |
|  | Diptera | 0.09 ± 0.19 | 0.640 | 0.01 |
|  | Trichoptera | 0.04 ± 0.19 | 0.832 | 0.00 |
|  | Ephemeroptera | 0.16 ± 0.19 | 0.401 | 0.03 |
|  | Neuroptera | −0.35 ± 0.18 | 0.060 | 0.12 |
|  | Coleoptera | −0.15 ± 0.19 | 0.443 | 0.02 |
|  | Hymenoptera | −0.19 ± 0.19 | 0.315 | 0.04 |
|  | Hemiptera | −0.15 ± 0.19 | 0.436 | 0.02 |
|  | Orthoptera | 0.20 ± 0.19 | 0.279 | 0.04 |

The sample sizes are 30. The predictors represent the relative abundance of different insects. Data with significant differences are noted in bold.
