# Supplementary figures and images for: Social calls influence the foraging behavior in wild big-footed myotis
Source: Front Zool. 2021 Jan 7;18:3. doi: 10.1186/s12983-020-00384-8 (PMC7791762; doi:10.1186/s12983-020-00384-8)

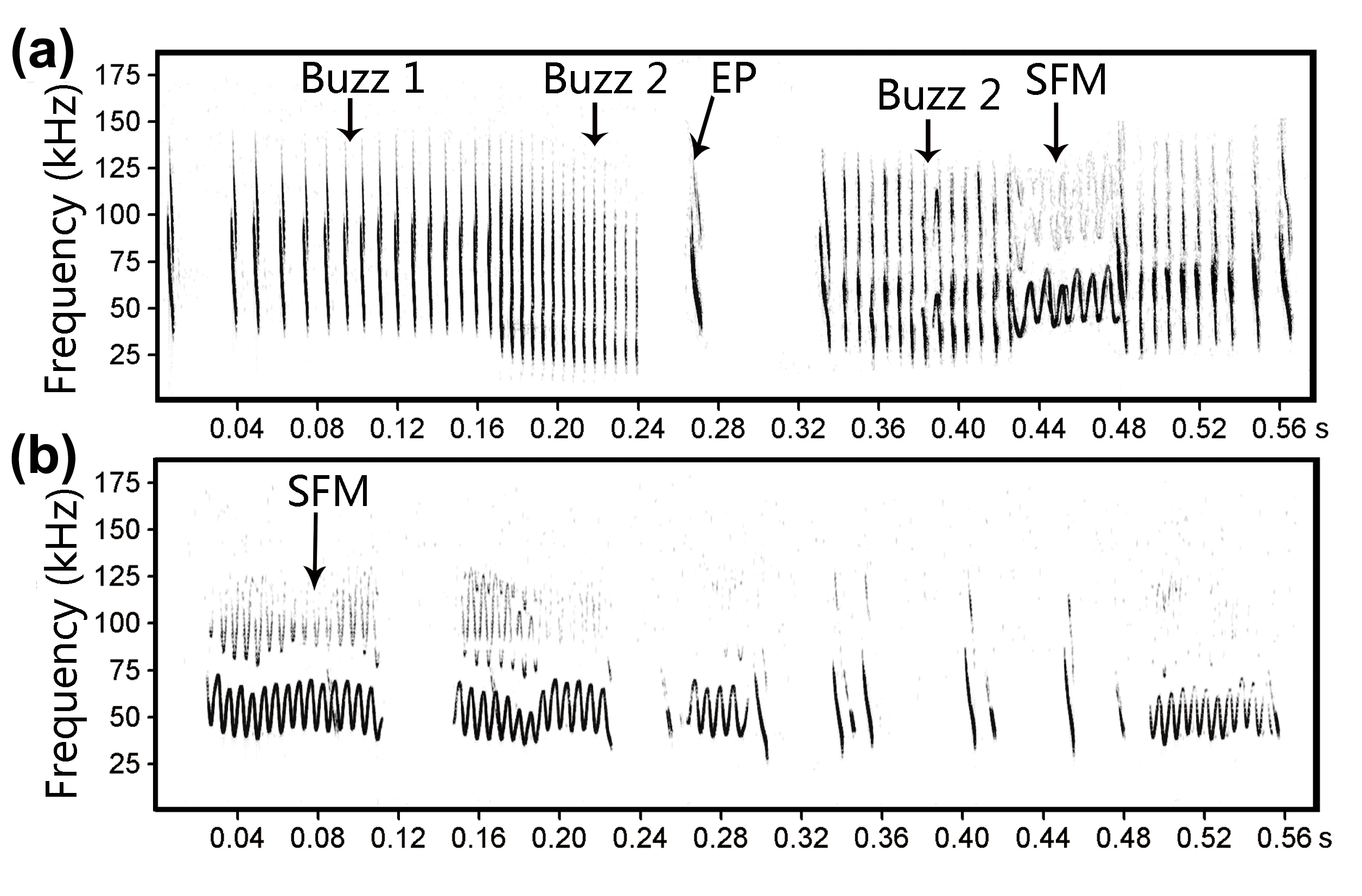

Supplement: Supplementary file 7 — Additional file 7: Figure S1. Spectrograms of echolocation pulses and sinusoidal frequency-modulated (SFM) calls. There was an overlap between the SFM type and other bats’ terminal buzzes. [file 12983_2020_384_MOESM7_ESM.jpg]

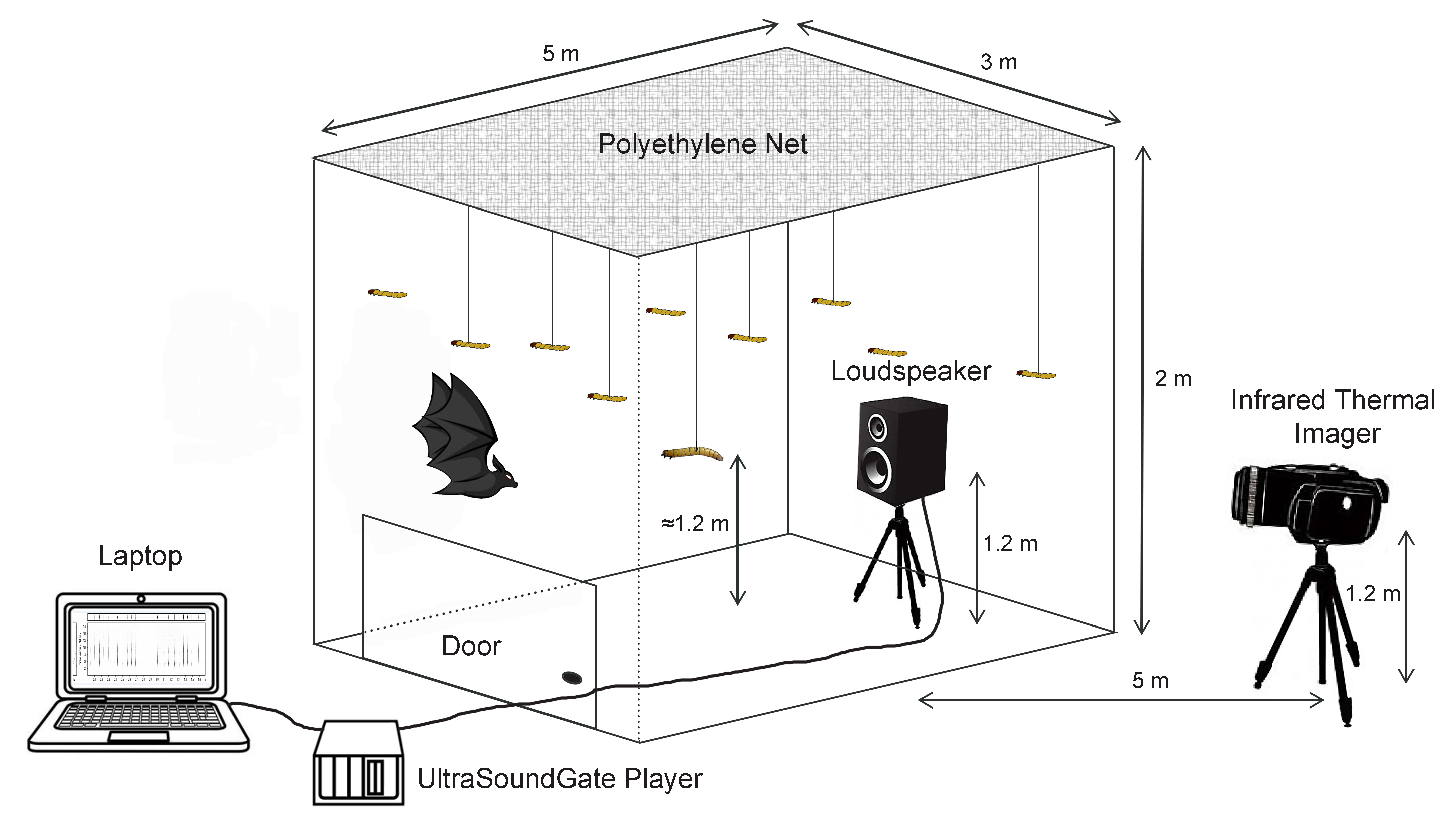

Supplement: Supplementary file 9 — Additional file 9: Figure S2. Experimental setup for playback experiments. [file 12983_2020_384_MOESM9_ESM.jpg]
