# Supplementary material for: Social calls influence the foraging behavior in wild big-footed myotis
Source: Front Zool. 2021 Jan 7;18:3. doi: 10.1186/s12983-020-00384-8 (PMC7791762; doi:10.1186/s12983-020-00384-8)
Supplement: Supplementary file 8 — Additional file 8: Table S7. Foraging activity in big-footed myotis across different transects. [file 12983_2020_384_MOESM8_ESM.docx]

**Table S7**

Foraging activity in big-footed myotis across different transects

| Transects | N_pulses_ | | | N_buzzes_ | | |
| --- | --- | --- | --- | --- | --- | --- |
|  | Group 1 | Group 2 | Group 3 | Group 1 | Group 2 | Group 3 |
| T1 | 0 | 3 | 0 | 0 | 0 | 0 |
| T2 | 1 | 1 | 0 | 0 | 0 | 0 |
| T3 | 2 | 8 | 3 | 0 | 0 | 0 |
| T4 | 38 | 46 | 23 | 9 | 11 | 6 |
| T5 | 9 | 1 | 7 | 0 | 0 | 0 |
| T6 | 0 | 0 | 16 | 0 | 0 | 1 |
| T7 | 6 | 19 | 8 | 0 | 0 | 0 |
| T8 | 3 | 24 | 19 | 0 | 1 | 0 |
| **T9** | **120** | **103** | **118** | **20** | **7** | **12** |
| T10 | 8 | 0 | 1 | 0 | 0 | 0 |
| T11 | 49 | 66 | 54 | 7 | 15 | 2 |
| T12 | 0 | 0 | 0 | 0 | 0 | 0 |
| T13 | 13 | 5 | 0 | 0 | 0 | 0 |
| T14 | 29 | 14 | 39 | 2 | 0 | 8 |
| T15 | 0 | 7 | 3 | 0 | 0 | 0 |

N_pulses_: the number of echolocation pulses; N_buzzes_: the number of feeding buzzes. The most commonly used transect is noted in bold.
