# Supplementary material for: Social calls influence the foraging behavior in wild big-footed myotis
Source: Front Zool. 2021 Jan 7;18:3. doi: 10.1186/s12983-020-00384-8 (PMC7791762; doi:10.1186/s12983-020-00384-8)
Supplement: Supplementary file 10 — Additional file 10: Table S8. The performance of each focal bat during the different playback trials [file 12983_2020_384_MOESM10_ESM.docx]

**Table S8**

The performance of each focal bat during the different playback trials

| Types of signals | Identity of bat | Number of trials | Food consumption | Flight duration |
| --- | --- | --- | --- | --- |
| Silence | No.1 | 4 | 16.4 ± 0.8 | 564.5 ± 49.0 |
| Silence | No.2 | 3 | 15.0 ± 0.0 | 541.0 ± 19.3 |
| Silence | No.3 | 3 | 15.7 ± 0.3 | 493.0 ± 82.4 |
| Silence | No.4 | 4 | 15.0 ± 1.2 | 493.5 ± 66.0 |
| Silence | No.5 | 4 | 12.5 ± 0.2 | 376.0 ± 40.3 |
| Silence | No.6 | 3 | 16.0 ± 0.0 | 568.0 ± 13.9 |
| Silence | No.7 | 4 | 16.3 ± 0.8 | 538.8 ± 24.1 |
| Silence | No.8 | 3 | 15.3 ± 0.9 | 519.3 ± 36.0 |
| Silence | No.9 | 4 | 16.4 ± 1.1 | 423.0 ± 9.4 |
| Silence | No.10 | 4 | 15.6 ± 1.2 | 381.8 ± 52.0 |
| Silence | No.11 | 4 | 17.0 ± 0.7 | 536.8 ± 46.9 |
| Silence | No.12 | 3 | 14.2 ± 0.7 | 544.0 ± 50.6 |
| Silence | No.13 | 3 | 14.7 ± 1.2 | 482.0 ± 54.1 |
| Silence | No.14 | 4 | 14.8 ± 0.5 | 512.5 ± 35.6 |
| EP | No.1 | 2 | 15.0 ± 2.0 | 440.5 ± 52.5 |
| EP | No.2 | 1 | 16.0 ± 0.0 | 554.0 ± 0.0 |
| EP | No.3 | 2 | 13.0 ± 1.0 | 415.0 ± 134.0 |
| EP | No.4 | 3 | 17.2 ± 1.6 | 507.0 ± 53.6 |
| EP | No.5 | 2 | 17.8 ± 1.8 | 483.0 ± 194.0 |
| EP | No.6 | 2 | 19.0 ± 0.0 | 659.5 ± 52.5 |
| EP | No.7 | 2 | 18.0 ± 1.0 | 538.5 ± 68.5 |
| EP | No.8 | 2 | 17.5 ± 0.5 | 552.0 ± 34.0 |
| EP | No.9 | 2 | 16.5 ± 0.5 | 408.0 ± 49.0 |
| EP | No.10 | 2 | 19.8 ± 0.3 | 621.0 ± 71.0 |
| EP | No.11 | 3 | 18.3 ± 0.7 | 528.7 ± 32.3 |
| EP | No.12 | 4 | 16.1 ± 1.1 | 531.3 ± 52.5 |
| EP | No.13 | 2 | 18.0 ± 1.0 | 444.0 ± 45.0 |
| EP | No.14 | 1 | 16.0 ± 0.0 | 511.0 ± 0.0 |
| bDFM | No.1 | 2 | 10.5 ± 0.5 | 320.0 ± 7.2 |
| bDFM | No.2 | 2 | 9.0 ± 0.0 | 209.0 ± 66.0 |
| bDFM | No.3 | 2 | 11.5 ± 3.5 | 255.0 ± 88.0 |
| bDFM | No.4 | 3 | 8.7 ± 2.2 | 208.3 ± 17.8 |
| bDFM | No.5 | 4 | 11.5 ± 0.5 | 237.3 ± 9.6 |
| bDFM | No.6 | 2 | 9.0 ± 1.0 | 236.5 ± 9.5 |
| bDFM | No.7 | 2 | 14.0 ± 0.0 | 297.5 ±16.5 |
| bDFM | No.8 | 3 | 9.0 ± 2.0 | 232.7 ± 38.6 |
| bDFM | No.9 | 2 | 12.5 ± 0.5 | 308.5 ± 14.5 |
| bDFM | No.10 | 2 | 13.3 ± 0.3 | 276.0 ± 9.0 |
| bDFM | No.11 | 2 | 12.5 ± 0.5 | 402.0 ± 98.0 |
| bDFM | No.13 | 2 | 10.0 ± 1.0 | 222.0 ± 33.0 |
| bDFM | No.14 | 2 | 13.3 ± 0.3 | 276.0 ± 23.0 |
| SFM | No.1 | 2 | 9.5 ± 2.5 | 394.0 ± 37.0 |
| SFM | No.2 | 2 | 9.5 ± 0.5 | 376.0 ±10.0 |
| SFM | No.3 | 2 | 9.0 ± 1.0 | 363.0 ± 80.0 |
| SFM | No.4 | 2 | 10.5 ± 1.5 | 347.5 ± 125.5 |
| SFM | No.5 | 2 | 8.5 ± 2.5 | 310.5 ± 162.5 |
| SFM | No.6 | 3 | 8.3 ± 1.3 | 378.3 ± 71.3 |
| SFM | No.7 | 2 | 11.5 ± 0.5 | 482.5 ± 10.5 |
| SFM | No.8 | 2 | 11.3 ± 1.3 | 434.5 ± 52.5 |
| SFM | No.9 | 2 | 9.5 ± 0.5 | 278.0 ± 56.0 |
| SFM | No.10 | 3 | 10.5 ±1.3 | 253.7 ± 55.6 |
| SFM | No.11 | 2 | 9.0 ± 1.0 | 376.5 ± 22.5 |
| SFM | No.12 | 2 | 10.8 ± 1.8 | 564.0 ± 39.0 |
| SFM | No.13 | 2 | 10.0 ± 1.0 | 500.5 ± 29.5 |
| SFM | No.14 | 2 | 7.0 ± 3.0 | 386.5 ± 83.5 |
| wDFM | No.1 | 2 | 8.8 ± 2.8 | 211.5 ± 103.5 |
| wDFM | No.2 | 2 | 8.5 ± 0.5 | 294.0 ± 10.0 |
| wDFM | No.3 | 2 | 11.0 ± 2.0 | 350.0 ± 71.0 |
| wDFM | No.4 | 2 | 12.5 ± 0.5 | 317.5 ± 90.5 |
| wDFM | No.5 | 2 | 6.5 ± 1.5 | 253.0 ± 30.0 |
| wDFM | No.6 | 2 | 9.3 ± 3.3 | 267.0 ± 84.0 |
| wDFM | No.7 | 3 | 9.7 ± 0.3 | 293.3 ± 24.7 |
| wDFM | No.8 | 2 | 10.0 ± 2.0 | 300.5 ± 96.5 |
| wDFM | No.9 | 2 | 10.5 ± 0.5 | 325.0 ± 7.0 |
| wDFM | No.10 | 2 | 10.0 ± 1.0 | 274.0 ± 75.0 |
| wDFM | No.11 | 2 | 10.0 ± 1.0 | 231.0 ± 17.0 |
| wDFM | No.12 | 2 | 11.5 ± 1.5 | 364.5 ± 11.5 |
| wDFM | No.13 | 3 | 11.3 ± 0.9 | 337.0 ± 50.1 |
| wDFM | No.14 | 2 | 14.0 ± 1.0 | 377.0 ± 35.0 |

Values are given as mean ± SE. Silence: silence control. EP: echolocation pulses. Food consumption: the number of consumed provisioned mealworms; Flight duration: time spent in flight (seconds).
